# Supplementary material for: Morpho-agronomic variability of okra [Abelmoschus esculentus (L.) Moench] genotypes in Dire Dawa, eastern Ethiopia
Source: PLoS One. 2023 Jul 14;18(7):e0288534. doi: 10.1371/journal.pone.0288534 (PMC10348553; doi:10.1371/journal.pone.0288534)
Supplement: Attachment — Submitted filename: PONE-D-23-10456 (2).pdf [file pone.0288534.s002.pdf]

PLOS ONE

Morpho-Agronomic Variability of Okra [Abelmoschus Esculentus (L.) Moench] Genotypes of Diverse Origin in Dire Dawa, Eastern Ethiopia

--Manuscript Draft--

**Manuscript Number:**	PONE-D-23-10456
**Article Type:**	Research Article
**Full Title:**	Morpho-Agronomic Variability of Okra [Abelmoschus Esculentus (L.) Moench] Genotypes of Diverse Origin in Dire Dawa, Eastern Ethiopia
**Short Title:**	Morpho-Agronomic Variability of Okra
**Corresponding Author:**	wubadis kenaw<br>Haramaya University College of Agricultural and Environmental Sciences<br>Harar, Oromiya (Oromia) ETHIOPIA
**Keywords:**	Key words: Cluster, Euclidean distances, Green fruit, Principal component axis and Variability.
**Abstract:**	Only a few studies were carried out to assess okra genotype variability and performance throughout the country. Therefore, it was necessary to continue the evaluation of available varieties to identify superior okra genotypes. Thus, this research was conducted to assess the morpho-agronomic and genetic variability of indigenous and introduced okra genotypes of at diverse origins. A total of 21 okra genotypes were evaluated for 25 morpho-agronomic traits in 2020 at Dire Dawa in a randomized complete block design with three replications. Analysis of variance showed significant differences for all traits. Estimates of genotypic (GCV) and phenotypic (PCV) coefficients of variation range from 9.16 to 42.3% and 9.33 to 44.16%, respectively. Heritability in broad sense (H2) and genetic advance as percent of mean (GAM) ranged from 29.57 to 91.89% and 10.39 to 83.53%, respectively. Estimated variability components (GCV, PCV, H2 and GAM) were high and moderate for all traits except days to 50% emergence (GCV and PCV), internode length (GCV) and green fruit width (H2) that were categorized under low. The first four principal component axes (PCA1 to PCA4) accounted for 7.83 to 35.02% that accounted 74.56% of the total variability with eigenvalues that ranged from 1.95 to 8.75. Genetic distances estimated by Euclidean distance from the 25 traits ranged from 2.33 to 12.56 with mean 6.83, standard deviation 1.8, and coefficient of variation 26.46%. The genotypes were grouped into four distinct clusters using Euclidean distance matrix using UPGMA. Indigenous okra genotypes collected from Ethiopia were more divergent with high genetic distances and had higher performance for most of the traits including growth, green fruits yield, and seed yield than introduced genotypes. In conclusion, this study showed the presence of variation among genotypes for most of the traits, indicating that selection of genotypes could be effective to develop okra varieties with high green fruit and seed yield through direct selection or crossing.
**Order of Authors:**	wubadis kenaw
	Wassu Mohammed
	Kebede Woldetsadik
**Additional Information:**	
**Question**	**Response**
**Financial Disclosure**<br><br>Enter a financial disclosure statement that describes the sources of funding for the work included in this submission. Review the submission guidelines for detailed requirements. View published research articles from *PLOS ONE* for specific	The author(s) received no specific funding for this work.

Powered by Editorial Manager® and ProduXion Manager® from Aries Systems Corporation

examples.<br><br>This statement is required for submission and **will appear in the published article** if the submission is accepted. Please make sure it is accurate.<br><br>**Unfunded studies**<br>Enter: *The author(s) received no specific funding for this work.*<br><br>**Funded studies**<br>Enter a statement with the following details:<br>• Initials of the authors who received each award<br>• Grant numbers awarded to each author<br>• The full name of each funder<br>• URL of each funder website<br>• Did the sponsors or funders play any role in the study design, data collection and analysis, decision to publish, or preparation of the manuscript?<br>• **NO** - Include this sentence at the end of your statement: *The funders had no role in study design, data collection and analysis, decision to publish, or preparation of the manuscript.*<br>• **YES** - Specify the role(s) played.<br><br>* typeset	
**Competing Interests**<br><br>Use the instructions below to enter a competing interest statement for this submission. On behalf of all authors, disclose any competing interests that could be perceived to bias this work—acknowledging all financial support and any other relevant financial or non-financial competing interests.<br><br>This statement is required for submission and **will appear in the published article** if the submission is accepted. Please make sure it is accurate and that any funding sources listed in your Funding Information later in the submission form are also declared in your Financial Disclosure statement.	The authors have declared that no competing interests exist.

View published research articles from *PLOS ONE* for specific examples.<br><br>**NO authors have competing interests**<br><br>Enter: *The authors have declared that no competing interests exist.*<br><br>**Authors with competing interests**<br><br>Enter competing interest details beginning with this statement:<br><br>*I have read the journal's policy and the authors of this manuscript have the following competing interests: [insert competing interests here]*<br><br>* typeset	
**Ethics Statement**<br><br>Enter an ethics statement for this submission. This statement is required if the study involved:<br><br>• Human participants<br>• Human specimens or tissue<br>• Vertebrate animals or cephalopods<br>• Vertebrate embryos or tissues<br>• Field research<br><br>Write "N/A" if the submission does not require an ethics statement.<br><br>General guidance is provided below. Consult the submission guidelines for detailed instructions. **Make sure that all information entered here is included in the Methods section of the manuscript.**	N/A

Powered by Editorial Manager® and ProduXion Manager® from Aries Systems Corporation

**Format for specific study types**<br><br>**Human Subject Research (involving human participants and/or tissue)**<br>• Give the name of the institutional review board or ethics committee that approved the study<br>• Include the approval number and/or a statement indicating approval of this research<br>• Indicate the form of consent obtained (written/oral) or the reason that consent was not obtained (e.g. the data were analyzed anonymously)<br><br>**Animal Research (involving vertebrate animals, embryos or tissues)**<br>• Provide the name of the Institutional Animal Care and Use Committee (IACUC) or other relevant ethics board that reviewed the study protocol, and indicate whether they approved this research or granted a formal waiver of ethical approval<br>• Include an approval number if one was obtained<br>• If the study involved *non-human primates*, add *additional details* about animal welfare and steps taken to ameliorate suffering<br>• If anesthesia, euthanasia, or any kind of animal sacrifice is part of the study, include briefly which substances and/or methods were applied<br><br>**Field Research**<br><br>Include the following details if this study involves the collection of plant, animal, or other materials from a natural setting:<br>• Field permit number<br>• Name of the institution or relevant body that granted permission	
**Data Availability**<br><br>Authors are required to make all data underlying the findings described fully available, without restriction, and from the time of publication. PLOS allows rare exceptions to address legal and ethical concerns. See the PLOS Data Policy and FAQ for detailed information.	Yes - all data are fully available without restriction

A Data Availability Statement describing where the data can be found is required at submission. Your answers to this question constitute the Data Availability Statement and **will be published in the article**, if accepted.<br><br>**Important:** Stating 'data available on request from the author' is not sufficient. If your data are only available upon request, select 'No' for the first question and explain your exceptional situation in the text box.<br><br>Do the authors confirm that all data underlying the findings described in their manuscript are fully available without restriction?	
**Describe where the data may be found in full sentences. If you are copying our sample text, replace any instances of XXX with the appropriate details.**<br><br>• If the data are **held or will be held in a public repository**, include URLs, accession numbers or DOIs. If this information will only be available after acceptance, indicate this by ticking the box below. For example: *All XXX files are available from the XXX database (accession number(s) XXX, XXX.*).<br>• If the data are all contained **within the manuscript and/or Supporting Information files**, enter the following: *All relevant data are within the manuscript and its Supporting Information files.*<br>• If neither of these applies but you are able to provide **details of access elsewhere**, with or without limitations, please do so. For example:<br><br>*Data cannot be shared publicly because of [XXX]. Data are available from the XXX Institutional Data Access / Ethics Committee (contact via XXX) for researchers who meet the criteria for access to confidential data.*<br><br>*The data underlying the results presented in the study are available from (include the name of the third party*	All relevant data are within the manuscript and its Supporting Information files.

and contact information or URL).<br>• This text is appropriate if the data are owned by a third party and authors do not have permission to share the data.<br><br>* typeset	
Additional data availability information:	

Manuscript
Click here to access/download;Manuscript;okra research manuscript PO.docx

Morpho-Agronomic Variability of Okra [*Abelmoschus Esculentus* (L.) Moench] Genotypes of Diverse Origin in Dire Dawa, Eastern Ethiopia

Wubadis Kenaw,[*1] Wassu Mohammed, [1] Kebede Woldetsadik[1]

[1,] School of Plant Sciences, Haramaya University 138 Dire Dawa, Ethiopia

Correspondence: Wubadis Kenaw
Email: wubadiskenaw2009@gmail.com or Wubadis.Kenaw@haramaya.edu.et

ABSTRACT

Only a few studies were carried out to assess okra genotype variability and performance throughout the country. Therefore, it was necessary to continue the evaluation of available varieties to identify superior okra genotypes. Thus, this research was conducted to assess the morpho-agronomic and genetic variability of indigenous and introduced okra genotypes of at diverse origins. A total of 21 okra genotypes were evaluated for 25 morpho-agronomic traits in 2020 at Dire Dawa in a randomized complete block design with three replications. Analysis of variance showed significant differences for all traits. Estimates of genotypic (GCV) and phenotypic (PCV) coefficients of variation range from 9.16 to 42.3% and 9.33 to 44.16%, respectively. Heritability in broad sense (H^2) and genetic advance as percent of mean (GAM) ranged from 29.57 to 91.89% and 10.39 to 83.53%, respectively. Estimated variability components (GCV, PCV, H^2 and GAM) were high and moderate for all traits except days to 50% emergence (GCV and PCV), internode length (GCV) and green fruit width (H^2) that were categorized under low. The first four principal component axes (PCA1 to PCA4) accounted for 7.83 to 35.02% that accounted 74.56% of the total variability with eigenvalues that ranged from 1.95 to 8.75. Genetic distances estimated by Euclidean distance from the 25 traits ranged from 2.33 to 12.56 with mean 6.83, standard deviation 1.8, and coefficient of variation 26.46%. The genotypes were grouped into four distinct clusters using Euclidean distance matrix using UPGMA. Indigenous okra genotypes collected from Ethiopia were more divergent with high genetic distances and had higher performance for most of the traits including growth, green fruits yield, and seed yield than introduced genotypes. In conclusion, this study showed the presence of variation among genotypes for most of the traits, indicating that selection of genotypes could be effective to develop okra varieties with high green fruit and seed yield through direct selection or crossing.

Keywords: Cluster, Euclidean distances, Green fruit, Principal component axis and Variability.

1. INTRODUCTION

Okra [*Abelmoschus esculentus* (L.) Moench] is one of the most widely utilized species of the family *Malvaceae* (Naveed, Khan and Khan, 2009). It is originated in Ethiopia and native in the area of Ethiopia and Sudan (Reddy *et al.*, 2012). It is cultivated as a vegetable crop in tropical, subtropical, warm, and temperate regions of the world (Lim, 2012). It is an important crop in most developing countries, covering approximately 4% of the total vegetable consumption, and it ranks above other vegetable crops including cabbage, amaranths, and lettuce (Mihretu, Weyessa and Adugna, 2014; Gemede *et al.*, 2015).

Okra is mainly grown for its immature pods that are consumed as a fresh vegetable, a snack, a cooked vegetable, or as an additive for soups, salads, and stews. It is a good source of carbohydrates, protein, dietary fibre, calcium, magnesium, potassium, vitamins A and C, fat, iron, thiamine, nicotinamide and riboflavin (Gemede *et al.*, 2015). Okra seed is an excellent source of

zinc, and rich in phenolic compounds, has a balanced protein of both lysine and tryptophan amino acids which is comparable to soybean (Adetuyi, Osagie and Adekunle, 2011). Seed flour are used to fortify cereal flour to increase protein, ash, oil, and fibre content in the meal. The potential for wide cultivation of edible oil as well as for cakes is very high because the oil content of the seed is high at about 40% (Mohammed *et al.*, 2022) and the oil is rich source of linoleic acid, a polyunsaturated fatty acid essential for human nutrition, rich in palmitic acid and oleic acids (Steyn *et al.*, 2014). The dried seeds can be used to prepare vegetable curds and used as a substitute and/or coffee additive after roasted. These properties enhance the importance of okra, pod, and seed foodstuff in the human diet (Arapitsas, 2008).

Okra is a perfect candidate for sustainable agriculture, the crop should be attractive to both producers and consumers in terms of its growth, yield, and quality. Considerably high magnitude of genetic variability of vigorous growth, earliness, and yield-associated traits have been reported in okra germplasm (YADAV *et al.*, 2017). This high genetic variability is a key to okra improvement. The existing variability has been exploited in various breeding programmes, which resulted in the development and release of a good number of varieties of okra. However, the released varieties cannot be continued longer due to the development and replacement of traditional crops by new high yielding varieties and the impact of climate change.

The first step for the improvement of indigenous vegetable crops is to study the genetic variability in the available genotypes. This is because the high genetic variability of genotypes gives a better opportunity for breeders to select directly for the traits of interest (Welsh, 1981).The genetic variability of okra landraces has been studied in Ethiopia (Binalfew and Alemu, 2016; Muluken, Wassu and Endale, 2016; Mohammed, Bekele and Kumar, 2017; YIMAM, 2018; Temam, Mohamed and Aklilu, 2020). The information generated from past studies gave a clue to the potential of improving okra genotypes. But the studies were focusing to assess the genetic diversity of okra genotypes for varied traits with respect to their diverse geographical origin. However, information on genetic diversity alone is not sufficient for the improvement of okra landraces for yield and fruit quality. The improvement of the crop requires continuous evaluation of genotypes for fruit yield, quality, and other morpho-agronomic traits, comparing the potential of indigenous with introduced varieties to identify and release varieties with short period as possible and

evaluating available okra indigenous and introduced varieties for stress tolerance to ascertain the crop resilient to climate change.

Therefore, it was necessary to continue the evaluation of available okra indigenous and introduced varieties for yield, fruit quality, and traits related to stress tolerance to ascertain the genetic variability of the genotypes of yield and its components to estimate the genotype potential to withstand stresses such as high temperature and to identify superior okra genotypes to be used in breeding programs.

Thus, this research was initiated to achieve the following objectives.

- To assess growth and fruit yield variations among indigenous and introduced okra genotypes, and
- To assess the genetic variability of indigenous and introduced okra genotypes for morpho-agronomic traits.

2. MATERIALS AND METHODS

2.1. Description of the Study Site

The experiment was conducted at Tony farm the research site of Haramaya University at Dire Dawa from February to June 2020 under irrigation conditions. Dire Dawa lies between latitudes 9°36′N, 41°52′E and characterized by a warm and dry climate with a relatively low level of precipitation. It has an altitude of 1260 meters above sea level, a mean annual temperature of about 24.8°C, an average maximum temperature of 31.4°C and an average minimum temperature of about 18.2°C. The aggregate average annual rainfall is about 604mm and the annual relative humidity is 41.82% (Mohammed and Burga, 2015).

2.2. Experimental Materials and Design

A total of 21 okra genotypes, 11 okra genotypes collected from four geographic regions (western, south-western, north-western, and northern Ethiopia) known as the major growing regions of okra in the country and 10 commercial varieties introduced from India and USA were evaluated in the present study. The four major growing regions of okra are represented by 5 genotypes (240586, 240609, 240591, 245157, and 242443) and 4 (240209, 92203, 240207 and 242433) genotypes collected from 490 to 935 and 1050 to 1480 meter above sea level, respectively. The nine genotypes were collected from nine known districts such as Akobo (490m), Gambella, Akobo (630), SirbAbaye, Menge, Mandura, DigaLeka, Dibate and Asossa and two genotypes Humera 01

and (*Bamaya-Humera*) collected from northern Ethiopia. Two (SOH 714 and SOH 701) introduced and registered commercial varieties by companies, seven commercial varieties introduced from India and one commercial variety introduced from USA for research purposes were used for the study (Table 1).

The field experiment was conducted in a randomized complete block design with three replications. Each genotype was randomly assigned to a plot in each replication in the experimental field. Each plot consisted of 12 plants at a spacing of 0.6 m between plants. The spacing between the plots and adjacent replications was 0.8 and 2 m, respectively. Three seeds per hill was sown and thinned to one plant per hill when plants reached 3-4 leaf stages.

Table 1. List of 21 Okra genotypes and their origin

No	Accession code	Regional state	District	Altitude m.a.s.l.	Geographic region
1	240586	Gambella	Akobo	490	Southwestern
2	240609	Gambella	Gambella	730	Southwestern
3	240591	Gambella	Akobo	630	Southwestern
4	240209	BenishangulGumuz	Mandura	1050	North-western
5	240207	BenishangulGumuz	Dibate	1400	North-western
6	92203	Oromia	DigaLeka	1200	Western
7	242433	BenishangulGumuz	Asossa	1480	Western
8	245157	BenishangulGumuz	SirbAbaye	870	Western
9	242443	BenishangulGumuz	Menge	935	Western
10	Humera 1	Tigray			Northern
11	23793 (Bamaya-Humera)	Tigray	Released variety		Northern
12	SOH 714		Registered commercial variety		
13	SOH 701		Registered commercial variety		
14	Dhenu				India
15	Kiran				India
16	Kraft				India
17	Pocha				India
18	Mithra				India
19	Arka Anamica				India
20	NamdHari				India
21	Clemson Spineless				USA

Source of genotypes: Haramaya University and Institute of Biodiversity and Conservation Research, Addis Ababa

2.3. Experimental Procedure and Plot Management

The seeds were harvested in January and February 2019 cropping season at Mellkasa Agriculture Research Centre and kept in canvas bags after drying and stored at Institute of Biodiversity and Conservation Research, Addis Ababa.

Land preparation was done in the first week of February 2020 using a tractor and human labour. The soil was levelled to permit furrow irrigation every five days. The rows were raised to increase soil surface area, aeration, and drainage. The ridges were made according to the plant spacing's by hand. Okra seeds were sown in mid-February 2020 and placed at the depth of 5 cm. Furrow irrigation was applied every five days. Cultural practices such as cultivation, hoeing, weeding, and earthing-up were applied uniformly. Green fruits were harvested every five days from 10 plants in the middle row to estimate green fruit yield and related traits and dry pods (green colour of pods changed to grey colour) from two plants at both ends of the row were harvested to collect seed yield and seed yield related data.

2.4. Data Collection

International Plant Genetic Resources Institute (IPGRI, 1991) descriptor list for okra species was used to record data on quantitative traits. Quantitative traits were recorded from ten plants per row/genotype, leaving the two plants grown at both ends of the row as boarder plants and to use for data collection for seed and related traits.

2.4.1. Crop Phenology

Days to 50% emergence: It was registered by counting the number of days from sowing to the emergence of 50% of seedlings from ten plants in each plot.

Days to first flowering: the number of days was taken from the date of sowing to the onset of the first flower appears for ten plants in each plot.

Days to 50% flowering: It was recorded by counting the number of days from planting to flowering of 50% of plants in each plot.

Days to maturity: the number of days from emergence of seedlings to the stage when 90% of the plants in a plot have reached physiological maturity (when the fruit is bright green, the pod is fleshy).

2.4.2. Growth Traits

Plant height (cm): the height of the plants from the ground surface to the tip of the main stem was measured in centimeters at the final harvest and the mean height of ten plants was registered as the mean plant height in centimeters for statistical analysis.

Stem diameter (cm): the diameter of the stem at the basal region was measured at the final harvest using a standard graduated scale Vernier caliper. The diameter of ten plants was averaged to record the mean stem diameter in centimeters.

Number of branches per plant: the average number of branches of ten plants from each plot at final harvest was registered.

Number of internodes: the total number of internodes per plant was counted at final picking and the average of ten plants was calculated.

Internode's length (cm): the length of the internodes between the fifth and sixth node was measured at the time of maturity before the first tender fruit harvest from ten plants.

Leaf length (cm): The length of 10 leaves on the main stem from each plot was sampled randomly from the 9th and 11th nodes taken at the time of flowering was measured from the attachment of the base of the leaves and petiole to the tip of the leaves using a ruler from ten plants.

Leaf width (cm): The width of 10 leaves on the main stem from each plot was sampled randomly from the 9th and 11th nodes at the time of flowering from ten plants.

2.4.3. Fruit Characteristics and Green Fruit Yield

Number of green fruits per plant: the number of green pods of ten plants in each plot was counted and averaged over the sample plant.

Weight of green fruits per plant (g): the green pod of ten plants in each plot was harvested, counted, and weighed to estimate and register the weight of green fruits per plant.

Green fruit length (cm): the length of five green fruits from each harvest in each plot was measured and the average lengths were calculated and recorded.

Green fruit width (cm): the width of five green pods from each harvest in each plot was measured and the averages were registered.

Average green fruits weight (g): five green fruits from each harvest in each plot that were used to measure pod length and width was weighed using a sensitive balance and the average weight will be calculated and recorded accordingly.

Fruit yield per hectare (t ha^{-1}): It was estimated from the 20 plants tender fruit yield in each plot and was calculated from fruit yield per hectare.

2.4.4. Dry Pod, Seed Characteristics and Seed Yield

Number of dry pods per plant: the number of dry pods in each plot were counted and averaged over the sample plants.

Weight of dry pods per plant (g): the dry pods of two plants in each plot were harvested, counted, and weighed to estimate and register the weight of dry pods per plant.

Average dry pod weight (g): the weight of dry pods of two plants from each plot were divided by the number of dry pods and the average weight a single pod was registered.

Number of seeds per pod: the seeds of five dry pods from each genotype from each replication was extracted, counted, and average numbers of seeds per pod were calculated and registered.

Hundred seeds weight (g):100 seeds extracted from five mature turned to dry pods (green colour of pods changed to grey colour) from each genotype from each replication were weighted, oven dried for 24 hours at 105°C, the glass crucible was removed and transferred into a desiccator for cooling after which the seeds were weighed and adjusted to 10% moisture content.

Moisture content of seeds (%): it was estimated from 100 seeds extracted from five dry pods and oven dried as follows:

$$\text{Seeds moisture content (\%)} = \frac{(WFS - WDS) * 100}{\text{WFS}}$$

WFS = weight of fresh seed sample

WDS = weight of dried seed sample

Seed weight per pod (mg): it was estimated from the number of seeds per pod and 100 seeds weight adjusted to 10% moisture content as follows:

$$\text{Seeds weight per pod (mg)} = \frac{\text{NSP}\times 100\text{SW(mg)}}{100}$$

NSP = number of seeds per pod

100SW (mg) = 100 seed weight adjusted to 10% moisture content

Seed yield per plant (g): it was estimated from the number of dry pods per plant and seed weight per pod as follows:

$$\text{Seeds yield per plant (g)} = \frac{\text{NDP} \times \text{SWP (mg)}}{1000}$$

NDP = number of dry pods per plant

SWP (mg) = seed weight per pod adjusted to 10% moisture content

2.5. Data Analysis

2.5.1. Analysis of Variance and Descriptive Statistics

The quantitative data was subjected to analysis of variance (ANOVA). The data recorded as percentage were transformed before subjecting to analysis of variance (Gomez and Gomez, 1984). The comparison of mean performance of genotypes was conducted following the significance of mean squares using the least significant difference at 5% probability level. The trait that exhibited significant mean squares in general ANOVA was further subjected to genetic analyses as a description given in the subsequent subtitle. Descriptive statistics were used to describe qualitative data.

2.5.2. Phenotypic and Genotypic Variability

The variability was estimated by genotypic and phenotypic variances and coefficient of variation. The genotypic and phenotypic variance were estimated as proposed by (Burton and De Vane, 1953):

$$\sigma^2p = \sigma^2g + \sigma^2e$$

$$\sigma^2 g = \frac{Mg - Me}{r}$$

With $\sigma^2 p$ = phenotypic variance

$\sigma^2 g$ = Genotypic variance

$\sigma^2 e$ = Environmental (error) variance (Error mean square)

Mg = mean sum square of genotypes

Me = mean sum square of error and r = number of replications

As well, the phenotypic and genotypic coefficient of variation were estimated using the methods proposed by (Burton and De Vane, 1953):

Genotypic coefficient of variation, $GCV = \frac{\sqrt{\sigma^2 g}}{\bar{X}} * 100$

Phenotypic coefficient of variation, $PCV = \frac{\sqrt{\sigma^2 p}}{\bar{X}} * 100$

With $\bar{X}$ the population mean

2.5.3. Heritability and Genetic Advance

Broad sense heritability was calculated for each of the considered characters by the formula proposed by reference (Jain and Allard, 1960) and (Hill and Mackay, 2004):

$H^2 = \frac{\sigma^2 g}{\sigma^2 p} * 100$

Where, $\sigma^2 g$ = genotypic variance, $\sigma^2 p$= phenotypic variance,

$\sigma^2 p = \sigma^2 g + \sigma^2 e$

With $\sigma^2 e$ is the environmental (error) variance

Concerning the genetic advance (GA) in the broad sense expected under selection, assuming the selection intensity of 5%, it was computed using a formula suggested by reference (Jain and Allard, 1960):

GA = K (σ_P) H^2,

Where GA= Expected genetic advance, σ_P = the phenotypic standard deviation, H^2 = the heritability, K= Selection differential (K=2.06 at 5% selection intensity).

GA (as % of the mean) = $\frac{GA}{\bar{X}}$ *100

Where,$\bar{X}$ = population mean.

2.5.4. Principal Component Analysis

The morpho-agronomic traits that exhibited significant mean squares in general ANOVA were pre- standardized to means of zero and variance of unity before principal component analysis (PCA) was performed to avoid bias due to differences in measurement scales (Manly, 1986). After standardization, the data were subjected to principal component (PC) analysis to understand which trait(s) much contributed to the divergence or total variability of genotypes.

2.5.5. Genetic Distance and Clustering

The morpho-agronomic traits that exhibited significant mean squares in general ANOVA were used to estimate genetic distances and clustering of genotypes. Genetic distances of 21 okra genotypes were estimated using Euclidean distance (ED) calculated from quantitative traits after standardization (subtracting the mean value and dividing it by the standard deviation) as established by reference (Sneath, 1979) as follows:

$$EDjk = \sqrt{\sum_{i=1}^{n}\left(Xij - Xik\right)^{2}_{2}}$$

Where; EDjk = distance between genotypes j and k; xij and xik = phenotype trait values of the ith character for genotypes j and k, respectively; and n = number of phenotype traits used to calculate the distance. The distance matrices of phenotype traits were used to construct a dendrogram based on the Un-weighted Pair-group Method with Arithmetic Means (UPGMA). The results of cluster analysis were presented in the form of a dendrogram. In addition, the mean ED was calculated for each genotype by averaging of a particular genotype with the other 20 genotypes. The calculated average distance (ED) was used to estimate which genotype(s) is closest or distant to others.

3. RESULTS AND DISCUSSION

3.1. Analysis of Variance

3.1.1. Phenology and Growth Traits

The results of analysis of variance of 21 okra genotypes for crop phenology and growth traits showed significant difference. The results indicated that the presence of significant variations among genotypes for crop phenology and growth traits might be given a good opportunity for

breeders to select genotypes with varied crop maturity and better growth performance. (Mihretu, Weyessa and Adugna, 2014) and (Muluken, Wassu and Endale, 2016) reported the presence of highly significant differences among okra genotypes for crop phenology (days to 50% emergence, 50% flowering, and days to maturity) and growth traits (number of branches and internode length). The okra genotypes were collected from south-western, north-western and western parts of Ethiopia.

3.1.2. Green Fruit Yield and Yield Components

The results of analysis of variance of 21 okra genotypes for number of green fruits, weight of green fruit per plant, green fruit length, average green fruits weight, green fruit width and fruit yield per hectare showed significant difference. The results indicated that the presence of significant differences among okra genotypes for green fruit yield and yield component traits might be given a good opportunity for breeders to select high yielding and most preferable genotypes. (Mohammed, Bekele and Kumar, 2017) reported the presence of significant differences in the number of fruits per plant, green fruit weight, fruit length, and fruit width.

3.1.3. Dry Pod, Seed Characteristics and Seed Yield

The genotypes exhibited significant differences for number of dry pods, weight of dry pods per plant, average dry pod weight, number of seeds per pod, hundred seeds weight, seed moisture content, seed weight per pod and seed yield per plant**.** The results indicated that the presence of significant differences among okra genotypes for seed yield and related traits will help breeders for hybridization/ selection of traits of interest**.** (Muluken, Wassu and Endale, 2016; Mohammed, Bekele and Kumar, 2017; YIMAM, 2018; Temam, Mohamed and Aklilu, 2020) reported similar significant differences for number of pods per plant, weight of dry pods per plant, average weight of dry pods, number of seeds per pod, seed weight per pod, hundred seed weight and seed yield per plant among the genotypes evaluated.

3.2. Mean Performance of Okra Genotypes

3.2.1. Phonological Traits

The recently released variety 23793 (*Bamaya Humera*) and other genotypes collected from northern Ethiopia (Humera 01) were earlier to attain 50% emergence (9.33 days) followed by the two registered commercial varieties (SOH 701 and SOH 714) 9.66 days. The two registered

commercial varieties (SOH 701 and SOH 714) also had mean days to 1st flowering (54.65days), 50% flowering (69.85days) and days to maturity (83.85days). The two genotypes (*Bamaya Humera* and Humera 01) collected from northern Ethiopia also had 46.5, 55.35, and 67 mean days to 50% emergence, days to 1st flowering and days to maturity, respectively. The seven genotypes (Dhenu, Kiran, Kraft, Pocha, Mithra, ArkaAnamica, and NamdHari) introduced from India also showed early mean days to 1st flowering (46.98), days to 50% flowering (55.47days) and days to maturity (66.64). Genotypes collected from north-western Ethiopia (240209 and 240207) also showed days to 50% emergence (11.67 days), 1st flowering (58 days), 50% flowering (74.5 days) and days to maturity (92.33 days). Likewise, genotypes collected from south-western Ethiopia (240586, 240609, and 240591) had mean days to 50% emergence of (11.11days), 1st flowering (60), 50% flowering (69.56) and days to maturity (84.46).

The genotype introduced from USA (Clemson Spineless) also showed mean days to 50% emergence of (12), 1st flowering (53.3), 50% flowering (62.3), and days to maturity (78.3). Genotypes collected from western Ethiopia (242443, 92203, 245157, and 242433) showed lately mean 11, 59.75, 75.91and 96.5 days to attain 50% emergence, 1st and 50% flowering and days to maturity, respectively (Table 2). The genotypes showed wide range of variations for phonological traits. Okra genotypes were introduced from other countries and the two commercial varieties were early 1st and 50% flowering and days to maturity than okra genotypes collected from Ethiopia. This earliness might be an advantage in areas where the rainy season is short and the producer might help in capturing early market which fetches high price in markets and earn high income by supplying fruit early where fruits are not harvested from other genotypes under irrigated production. The research results suggested the higher chance of developing okra varieties for better growth, green fruit yield, and other desirable traits either through selection, breeding, and/or crossing of genotypes.

(Temam, Mohamed and Aklilu, 2020) reported significant variations for days to 50% emergence (7.5 to 11 days), days to first flowering (44.5 to 71 days), days to 50% flowering (48.5 to 77.5 days) and days to maturity (75.5 to 104.5 days). He also reported genotypes collected from western Ethiopia (Benishangul Gumuz) had mean performance greater than the mean values of the three tested genotypes for days to emergence. (Mihretu, Weyessa and Adugna, 2014) also observed 37 to 65 and 50 to101 days of first flowering and maturity, respectively, in 25 okra genotypes collected from south-western Ethiopia. This indicates early maturing okra genotypes can be useful

to escape drought conditions and can be cultivated as a climate change crops in drought prone areas in Ethiopia. (YIMAM, 2018) also reported variations for days to 50% flowering and days to maturity in the range between 64.18 to 89.84 and 73.33 to 105.67, respectively. Okra plant usually sets its first flower one to two months after sowing and has a maturity duration of 90 to100 days. (Muluken, Wassu and Endale, 2016; Melaku, Mohamed and Kumar, 2020) also reported introduced okra genotypes had the lowest days to 1st and 50% flowering and days to maturity in their finding's indicating the introduced genotypes were early compared to okra genotypes collected from Ethiopia.

3.2.2. Growth Traits

The genotype introduced from India (Dhenu, Kiran, Kraft, Pocha, Mithra, ArkaAnamica, and NamdHari) and the two registered commercial varieties (SOH 714 and SOH 701) and genotype introduced from USA (Clemson Spineless) and showed highest plant height 162.26, 173.30 and 180.08cm, respectively. Genotypes collected from north-western Ethiopia (240209 and 240207) and western (242443, 92203, 245157, and 242433) had mean of plant height 148.94 and 154.2cm, respectively. Genotypes collected from south-western (240586, 240609, and 240591) had 130.93cm and northern Ethiopia (*Bamaya Humera* and Humera 01) 137.02cm showed lowest plant height. The genotypes introduced from USA (Clemson Spineless) 3.39, north-western (240209 and 240207), 3.51cm, registered commercial varieties (SOH 701 and SOH 714) 3.61cm, western (242443, 92203, 245157 and 242433) 3.74cm and south-western Ethiopia (240586, 240609 and 240591) 3.77cm had widest stem diameter of while the remaining group of genotypes collected from northern Ethiopia (*Bamaya-Humera* and Humera 01) and genotypes introduced from India (Dhenu, Kiran, Kraft, Pocha, Mithra, ArkaAnamica and NamdHari) had narrowest stem diameter of 2.80 and 3.03, respectively. Genotypes introduced from USA, the two registered commercial varieties (SOH 701 and SOH 714), genotypes collected from south-western (240586, 240609, and 240591), north-western (240209 and 240207) and western Ethiopia (242443, 92203, 245157 and 242433) showed highest number of branches per plant 4.30, 4.53, 4.57, 5.05 and 5.31, respectively. While the remaining group of genotypes collected from northern Ethiopia and introduced from India showed 2.73 and 2.93, respectively (Table 2).

The two registered commercial varieties (SOH 701 and SOH 714), genotypes collected from north-western (240209 and 240207), south-western (240586, 240609 and 240591), western Ethiopia (242443, 92203, 245157 and 242433) and introduced from USA (Clemson Spineless) showed

mean values of longest leaf length 18.95, 19.61, 21.34, 21.37 and 22.16cm, respectively. While genotypes collected from northern Ethiopia *(Bamaya Humera* and Humera 01) introduced from India showed the shortest leaf length 16.51 and 17.38cm, respectively. The genotypes collected from north-western Ethiopia (240209 and 240207) 24.75cm, introduced from USA 25.73cm, western (242443, 92203, 245157 and 242433) 26.94cm and south western Ethiopia 27.79cm showed widest leaf width, while the remaining group of genotypes collected from northern Ethiopia (*Bamaya Humera* and Humera 01), introduced from India (Dhenu, Kiran, Kraft, Pocha, Mithra, ArkaAnamica and NamdHari) and the two registered commercial varieties (SOH 701 and SOH 714) showed leaf width 20.11, 21.16 and 22.56cm, respectively (Table 2**).**

The genotype introduced from USA (Clemson Spineless), 31.8, genotypes collected from western Ethiopia (242443, 92203, 245157, and 242433), 32.3, genotypes introduced from India (Dhenu, Kiran, Kraft, Pocha, Mithra, ArkaAnamica and NamdHari) 33.78 and two registered commercial varieties (SOH 701 and SOH 714) 35.16 had highest mean values for number of internodes per plant, while other group of genotypes collected from south western (240586, 240609 and 240591), north western (240209 and 240207) and northern Ethiopia (*Bamaya-Humera* and Humera 01) had lowest mean values for number of internodes per plant 27.28, 27.99 and 28.56, respectively. The genotypes collected from western (242443, 92203, 245157, and 242433), south-western (240586, 240609, and 240591), north-western Ethiopia (240209 and 240207) and introduced from USA (Clemson Spineless) showed the longest mean values of internode length 4.91, 4.95, 5.35 and 5.66cm, respectively, while other group of genotypes collected from northern Ethiopia, introduced from India and the two registered commercial varieties showed lower internodes length 4.79, 4.80 and 4.88cm, respectively (Table 2).

(YIMAM, 2018) observed significant variations among okra genotypes for plant height (175.2 cm), stem diameter (2.67cm), and internode length (8.99cm) traits. It was also reported okra genotypes collected from different growing regions of Ethiopia showed more variation in growth traits compared to genotypes introduced from other countries. (Mihretu, Weyessa and Adugna, 2014) observed significant variations among okra genotypes for plant height and stem diameter in the range between 42.96 to 96 cm and 2.2 to 3.9 cm, respectively. (Muluken, Wassu and Endale, 2016) also reported 110.5 to 302.13 cm and 1.97 to 3.72 cm of plant height and stem diameter, respectively. He also reported okra genotypes collected from Ethiopia had higher mean values than the two exotic okra varieties for stem diameter and number of branches.

Table 2. Mean values of 21 okra genotypes respect to geographic region for phenology and growth traits evaluated at Dire Dawa in 2020

Geographic region	Emergence (50%)	1st flowering	50% flowering	Days to maturity	Plant height	Stem diameter	Number of branches	Leaf length	Leaf width	Number of internodes	Internode length
India	11.33^{a}	46.99^{b}	55.47^{b}	66.64^{c}	162.26^{ab}	3.03^{bc}	2.93^{b}	17.38^{b}	21.16^{bc}	33.78^{ab}	4.80^{b}
Northern	9.33^{c}	46.50^{b}	55.35^{b}	67.00^{c}	137.02^{cd}	2.80^{c}	2.73^{b}	16.515^{b}	20.11^{c}	28.56^{c}	4.79^{b}
North-western	11.67^{a}	58.00^{a}	74.50^{a}	92.33^{ab}	148.94^{bcd}	3.51^{a}	5.05^{a}	19.61^{ab}	24.75^{ab}	27.995^{c}	5.35^{a}
RCVs	9.67^{bc}	54.65^{ab}	69.85^{a}	83.85^{b}	173.30^{a}	3.61^{a}	4.53^{a}	18.95^{ab}	22.56^{bc}	35.16^{a}	4.88^{b}
South-western	11.11^{ab}	60.00^{a}	69.57^{a}	84.47^{b}	130.93^{d}	3.77^{a}	4.57^{a}	21.34^{a}	27.79^{a}	27.28^{c}	4.95^{a}
USA	12.00^{a}	53.30^{ab}	62.3^{ab}	78.3^{bc}	180.08^{a}	3.39^{ab}	4.30^{a}	22.16^{a}	25.73^{ab}	31.80^{abc}	5.66^{a}
Western	11.00^{ab}	59.75^{a}	75.91^{a}	96.50^{a}	154.2^{bc}	3.74^{a}	5.31^{a}	21.37^{a}	26.94^{a}	32.30^{bc}	4.91^{a}

RCVs= Registered commercial variety, USA = United States of America

(Melaku, Mohamed and Kumar, 2020) reported the Ethiopian collections were superior over introduced genotypes for stem diameter, number of branches per plant, leaf length, and leaf width. (Binalfew and Alemu, 2016) reported there was wide range of variations in growth traits of okra genotypes collected from different regions of Ethiopia. Besides the existence of a wide range of variations in growth traits, the okra genotypes collected from Ethiopia were more vigorous than the two registered commercial varieties introduced from India. (Temam, Mohamed and Aklilu, 2020) also reported variation among okra genotypes in plant height that ranged between 73.05 to 194.5cm, stem diameter 14.5 to 30.5mm, number of branches 1.3 to 12.7, number of internodes 13.5 to 39 and internode length 2.5 to 10.25cm. (Gerrano, 2018) also reported number of branches that ranged from 3.83 to 10.44 in South Africa.

The result indicates that genotypes collected from south-western, north-western, northern, and western Ethiopian had higher mean performance values of growth traits except plant height and number of internodes than the introduced genotypes. Genotypes with long and wide leaves, and many number of internodes and long internode length might help for higher photosynthesis activity, increased plant height, and thereby contributed to produce higher fruit yield. Genotypes that have vigorous growth traits are mostly preferable to local farmers. In another way, tall plants lodge easily due to environmental factors such as excessive flooding, rain, and wind when they produce tall fruit during favourable growing seasons. However, they are also essential for firewood, construction of houses, fences and for livestock feed. Furthermore, selection for the tall height genes might be important when the yield performance is low during unfavourable environmental conditions. The genotype that had a tall gene had the highest mean values in the number of internodes, internode length, and thick stem. The thicker stem, resists the environmental influences from lodging and can withstand high fruit yield (Gerrano, 2018).

3.2.3. Green Fruit Yield and Yield Components

Genotypes collected from south western (240586, 240609, and 240591), northern Ethiopia (*Bamaya Humera* and Humera 01), introduced from USA (Clemson Spineless) and north-western (240209 and 240207) showed the highest mean value number of green fruits per plant 16.53, 18.23 19.23 and 19.81, respectively. On the other side, the two registered commercial verities (SOH 701 and SOH714), genotypes introduced from India (Dhenu, Kiran, Kraft, Pocha, Mithra, ArkaAnamica and NamdHari) and western (242443, 92203, 245157 and 242433) had produced smallest mean number of green fruits per plant 9.36, 13.33 and 15.13, respectively. Genotypes collected from south-western (336.41g), north western (361.8g), northern Ethiopia (414.44g), and introduced from USA (475.45g) had produced the highest mean values of green fruit weight, while the remaining group of genotypes, the two registered commercial varieties, western Ethiopia and introduced from India showed lowest mean values of weight of green fruit per plant 174.20, 263.34 and 264.16g, respectively (Table 3).

All groups of genotypes except genotypes introduced from USA (Clemson, Spineless) showed mean vales of green fruit length from 13.27 to 16.67cm. Moreover all groups of genotypes except genotypes collected from western Ethiopia had the highest mean values of green fruit width from 2.13 to 2.44cm. The longer and wider green fruits with higher green fruit weight are preferred by consumers (Bizuayehu *et al.*, 2017). The research results showed the presence of a higher chance for selection of genotypes for longer and wider green fruits with higher green fruit weight. The genotypes collected from northern Ethiopia (45.81g) and introduced from USA (49.47g) showed the highest mean values of average green fruit weight, while the two registered commercial varieties (SOH 701 and SOH 714), western, introduced from India, south western and north-western Ethiopia showed mean values of average green fruit weight 28.11, 33.48, 33.69, 35.82 and 36.32g, respectively. The genotypes collected from south-western, north-western, and northern Ethiopia and introduced from USA showed the highest mean of fruits yield 7, 7.53, 8.63 and 9.9 tons ha^{-1}, respectively. While the two registered commercial varieties (SOH 701 and SOH 714), genotypes collected from western Ethiopia and introduced from India (Dhenu, Kiran, Kraft, Pocha, Mithra, ArkaAnamica and NamdHari) showed the lowest mean of fruits yield 3.62, 5.48 and 5.5 tons ha^{-1}, respectively. The genotypes had a wide range of green fruit yield. This result may be due to the cumulative effect of the number of green fruits per plant, green fruit weight, average fruit

weight, and green fruit length showing the genetic response of genotypes to environmental conditions (Table 3).

Table 3. Mean values of 21 okra genotypes respect to geographic region for green fruit characteristics and fruit yield evaluated at Dire Dawa in 2020

Geographic region	No Green fruit per plant	Weight of Green Fruit per Plant (g)	Green Fruit Length (cm)	Green Fruit Width (cm)	Average Green Fruit weight(g)	Fruit yield (tons ha^{-1})
India	13.33^{c}	264.16^{bc}	14.84^{ab}	2.13^{ab}	33.69^{c}	5.50^{bc}
Northern	18.23^{ab}	414.44^{a}	13.27^{ab}	2.34^{a}	45.81^{ab}	8.63^{a}
North-western	19.81^{a}	361.80^{ab}	16.09^{ab}	2.19^{ab}	36.32^{bc}	7.53^{ab}
RCVs	9.36^{d}	174.20^{c}	16.67^{a}	2.14^{ab}	28.11^{c}	3.62^{c}
South-western	16.53^{ab}	336.41^{ab}	16.15^{ab}	2.13^{ab}	35.82^{c}	7.00^{ab}
USA	19.23^{ab}	475.45^{a}	10.21^{b}	2.44^{a}	49.47^{a}	9.90^{a}
Western	15.13^{bc}	263.34^{bc}	13.74^{ab}	1.86^{b}	33.48^{c}	5.48^{bc}

RCVs= Registered commercial variety, USA = United States of America

The current study result indicates that there is a wide variation in green fruit yield and yield component traits of okra genotypes collected from different parts of Ethiopia and introduced to other countries, which is a key solution to the current climate change concern (Binalfew and Alemu, 2016). Most of the genotypes collected from south-western, north-western, northern, and western Ethiopia had higher mean performance of green fruits yield and yield components than the introduced and two registered commercial varieties. This showed that there is wide variability in green fruit yield and yield components in Ethiopia through collection and selection of okra genotypes with the desired traits (Melaku, Mohamed and Kumar, 2020).

(Muluken, Wassu and Endale, 2016) observed 25 okra genotype and reported mean values of genotypes in the range of 25.65 to 71.67g, 6.95 to 20.73cm and 17.5 to 48.47 tons ha^{-1} for fruit weight, fruit length and fruit yield with a mean value of 50.02g, 14.86 cm and 30.38 tons ha^{-1}, respectively. He also reported Ethiopian okra genotypes had higher mean values than the two exotic okra genotypes for number of fruit per plant and fruit yield per hectare. (Binalfew and Alemu, 2016) reported means of genotypes ranging from 6.47 to 61.67g for average fruit weight indicating the variability among okra collections from Ethiopia. (Temam, Mohamed and Aklilu, 2020) also tested 36 okra genotypes and reported the average fruit weight and fruit yield per hectare of genotypes varied from 17.04 to 67.23g and 9.44 to 32.88tons, respectively.

3.2.4. Dry Pod, Seed Characteristics and Seed Yield

All groups of genotypes showed the highest mean number of dry pods per plant from 13.16 to 17.68. The genotypes introduced from USA (683.8), the two registered commercial varieties (721g), genotypes collected from western (771.26g), south western (895.43g) and north-western Ethiopia (897.57g) had higher mean values of dry pod weight without non significance difference among them, while the remaining group of genotypes introduced from India and collected from northern Ethiopia showed dry pod weight 534.57 and 594.65g, respectively. Genotypes collected from north-western, south-western, and western showed higher mean values of average dry pod weight per plant 51.06, 53.06, and 66.78g, while the other genotypes introduced from India, collected from northern Ethiopia, the two registered commercial varieties and introduced from USA showed mean values of average dry pod weight 34.11, 41.73, 42.73 and 45.7g, respectively (Table 4).

The two registered commercial varieties (SOH 701 and SOH 714), USA (Clemson Spineless), genotypes collected from western, south-western and north-western Ethiopia showed the highest mean values of number of seeds per pod 88.16, 88.53, 91.88, 93.57 and 96.3, respectively. While genotypes collected from northern Ethiopia (*Bamaya-Humera* and Humera 01), introduced from India (Dhenu, Kiran, Kraft, Pocha, Mithra, ArkaAnamica and NamdHari) had lowest mean values of number of seed per pod 33.33 and 69.88, respectively. Genotypes collected from western and south western Ethiopia showed the highest mean values of hundred seed weight 6.33 and 6.91g, respectively. While the remaining groups of genotypes collected from northern Ethiopia, introduced from USA (Clemson Spineless), the two registered commercial varieties, genotypes collected from north western Ethiopia and introduced from India showed the lowest mean values of hundred seed weight 3.46, 5.8, 6.06, 6.24 and 6.26g, respectively (Table 4). The current result indicate that the highest number of seeds and hundred seed weight were considered as selection criteria for the breeding of okra genotypes for seed yield and yield component traits (Gerrano, 2018).

Genotypes collected from northern Ethiopia (*Bamaya Humera* and Humera 01), 7.17% had the lowest mean values of seed moisture content, while the remaining group of genotypes collected from north western (240209 and 240207), western Ethiopia (242443, 9220, 245157 and 242433), two registered commercial varieties (SOH 701 and SOH 714) and introduced from USA (Clemson Spineless) had higher seed moisture content from 9.4 to 11.42%. Moisture content is an index of

its water activity important for stability in seeds. Low moisture content in the seed is important to extend the shelf-life of okra seeds in storage place since selecting genotypes that had low seed moisture content is one of the climate smart post-harvest handling technology to save farmer's economic loss. Genotypes introduced from USA (5168.9mg), two registered commercial verities (5386.9mg), genotypes collected from western (6005.3mg), north western (6045.9mg) and south western Ethiopia (6483.56mg) had highest mean values of seed weight per pod without non significance difference among them, while the remaining group genotypes collected from northern (*Bamaya-Humera* and Humera 01) and introduced from India showed lower mean of seed weight per pod 2032.5 and 4580.47mg, respectively. genotypes collected from south-western Ethiopia (60.66g), the two registered commercial verities (61.30g) and genotypes collected from northern-western (61.98g) had higher mean values of seed yield per plant, while genotypes collected from northern, western Ethiopia, introduced from USA and India showed lower mean values of seed yield per plant 14.72, 39.09, 40.38 and 41.51g, respectively (Table 4).

Table 4. Mean values of 21 okra genotypes respect to geographic region for seed yield and related traits evaluated at Dire Dawa in 2020.

Geographic region	No dry pod per plant	Weight of dry pod per Plant(g)	Average dry pod weight (g)	Number of seeds per pod	100 seeds weight (g)	Seed moisture content (%)	Seeds weight per pod (mg)	Seed yield per plant (g)
India	15.89[a]	534.57[c]	34.11[c]	69.88[b]	6.26[b]	10.34[a]	4580.47[b]	41.51[bc]
Northern	14.66[a]	594.65[bc]	41.73[bc]	33.33[c]	3.46[c]	7.17[b]	2032.50[c]	14.72[d]
North-western	17.66[a]	897.57[a]	51.06[ab]	96.30[a]	6.24[b]	10.20[a]	6045.90[a]	61.98[ab]
RCVs	17.68[a]	721.00[abc]	42.73[bc]	88.16[a]	6.06[b]	11.27[a]	5386.90[ab]	61.30[a]
South-western	17.05[a]	895.43[a]	53.06[ab]	93.57[a]	6.91[a]	9.40[a]	6483.56[a]	60.66[a]
USA	15.00[a]	683.80[abc]	45.70[bc]	88.53[a]	5.80[b]	11.42[a]	5168.90[ab]	40.38[bc]
Western	13.16[a]	771.26[ab]	66.78[a]	91.88[a]	6.33[ab]	10.74[a]	6005.30[a]	39.09[c]

RCVs= Registered commercial variety, USA = United States of America

The observed high number of dry pods, number of seeds per pod, and hundred seed weight in okra genotypes suggested that the higher chance of improving seed yield of okra to produce high amount of edible oil per unit area. The oil content of the seed is quite high at about 40% and the seeds are also used as a substitute for coffee (Mohammed *et al.*, 2022).

(YIMAM, 2018) reported genotypes had significantly different mean values for number of dry pods, weight of dry pods per plant, average dry pod weight, number of seeds per pod, seed weight per pod, hundred seed weight and seed yield per plant that ranged from 3.53 to 24.73, 175.9 to 1518.9 g, 12.55 to 83.93g, 110.17, 7.09g, 7.01g and 5.86 to 153.89g respectively. (Mohammed, Bekele and Kumar, 2017) also reported group of genotypes collected from Ethiopia had higher mean values than introduced genotypes for the number of dry or mature pods per plant, weight of dry or mature pods and hundred seed weight. (Temam, Mohamed and Aklilu, 2020) Reported genotypes had a wide range of variation 58.5 to 111.9, 5.08 to 7.16g, and 33.48 to 198.04g with mean values of 79.56, 5.93g and 99.7g for number of seeds per plant, hundred seed weight and seed yield per plant, respectively. (Muluken, Wassu and Endale, 2016) also reported okra genotypes collected from Ethiopia had the highest mean values for the number of seeds per pod and hundred seed weight. Genotypes collected from different growing parts of Ethiopia are promising to be developed to variety with high seed yield than introduced (Mihretu, Weyessa and Adugna, 2014; Binalfew and Alemu, 2016; Muluken, Wassu and Endale, 2016). This suggests the higher chance to improving seeds yield through selection among okra genotypes collected from Ethiopia to produce high amount of edible oil and for other purposes such as seeds as a substitute for coffee, protein source etc.

3.3. Estimates of Genetic Variability Components

3.3.1. Genotypic and Phenotypic Variability

The estimates of genotypic (GCV) and phenotypic (PCV) coefficients of variation for 25 quantitative traits of okra genotypes are presented in table 5. The estimates of GCV and PCV were in the range from 9.16 to 42.3% and 9.33 to 44.16%, respectively. According to (Sivasubramanian and Madhavamenon, 1973), GCV and PCV can be categorized as low (<10%), moderate (10-20%), and high (>20%). Correspondingly, days to 50% emergence, internode length and green fruit width had computed low GCV (<10%). Moderate GCV and PCV (10-20%) values were computed for growth traits (plant height, stem diameter, number of internodes, leaf length, and width) and hundred seed weight. Moderate GCV values were computed for phenology traits (days to 1st and 50% flowering and days to maturity) and seed moisture content.

Moderate PCV values were computed for internode length and green fruit width. Phenotypic coefficient of variation for days to 50% emergence was also computed less than 10%. High values of PCV were computed for phonological traits (days to 1st and 50% flowering and days to maturity) and seed moisture content. High GCV and PCV were computed for the number of branches, all green fruit yield and related traits except green fruit width, seed yield related traits such as hundred seed weight, and seed moisture content. Green fruit width was also showed moderate values of PCV. This result indicates that most of the traits were less influenced by environmental factors and selection based on phenotypic expression of the genotypes could be applied as a breeding method to improve the traits. Many researchers suggested consistent differences of okra genotypes due to genotype and environmental interactions (Mihretu, Weyessa and Adugna, 2014; Muluken, Wassu and Endale, 2016; YIMAM, 2018; Temam, Mohamed and Aklilu, 2020). Therefore, the presence of genetic variability in crops is essential for its further improvement, providing options for breeders to develop new varieties and hybrids and high phenotypic and genotypic coefficient of variation is an indication of the influence of environmental factors in the expression of traits and the higher chance to improve the best traits through selection breeding.

(YIMAM, 2018) computed and reported phenotypic and genotypic coefficients of variation that ranged from 8.35% to 57.70% and 5.44 to 49.20%, respectively. Low PCV and GCV values were computed for phenological traits (days to first flowering and 50% flowering) and moderate values

of both PCV and GCV for plant height. She also reported moderate values of GCV with high values of PCV for stem diameter and hundred seed weight. Furthermore, she reported high values of GCV and PCV for days to 50% emergence, number of branches, number of internodes per plant, internode length, weight of dry pods per plant, average dry pod weight, number of seeds per pod, seed weight per pod and seed yield per plant. (Temam, Mohamed and Aklilu, 2020) also reported that the genotypic and phenotypic coefficients of variation ranged from 6.3 to 54.19% and 9.19 to 55.51%, respectively, and reported low GCV and PCV for days to maturity and hundred seed weight. He also reported high GCV and PCV for number of branch, number of fruits per plant, internode length, average fruit weight, fruit length, and internode length. Furthermore, he also reported moderate values of PCV and GCV for days to 50% emergence, days to 1st and 50% flowering, plant height, stem diameter, and fruit width (10-20%).

(Muluken, Wassu and Endale, 2016) reported low GCV and PCV for days to 50% emergence, internode length and moderate GCV and PCV for days to 1st and 50% flowering and maturity, stem diameter, number of internodes, fruit width, number of seeds per pod and hundred seed weight. The author also indicated that both GCV and PCV were high for plant height, number of branches, and number of green fruits per plant and weight of dry pods. (Mohammed, Bekele and Kumar, 2017) also estimate values of GCV and PCV as low for phenology traits (days to 50% emergence, 50% flowering, and maturity), moderate GCV and PCV values for stem diameter, internode length, leaf length, leaf width, fruit width and hundred seed weight. Likewise, he reported high GCV and PCV values for fruit yield, number of branches per plant, number of dry pods per plant, fruit weight, and weight of dry pods. (Mihretu, Weyessa and Adugna, 2014) also reported high values of PCV and GCV for the number of branches per plant.

3.3.2. Estimates of Heritability and Genetic Advance

3.3.2.1. Estimates of broad sense heritability

Estimates of heritability in broad sense (H^2) for 25 quantitative traits of okra genotypes are presented in table 5. The heritability values in the broad sense ranged from 29.57 to 91.89%. According to (Johnson, Robinson and Comstock, 1955)), heritability is categorized as low (0-30%), moderate (31-60%), and high > 60%. Based on this classification, all phenological traits (days to 50% emergence, 1st and 50% flowering, and days to maturity), growth traits (stem diameter, number of internodes, internode length, leaf length and width), and seed moisture content

fall under the moderate heritability category (31-60%), while the other traits, plant height, number of branches per plant, number of green fruits, weight of green fruits, green fruit length, average fruit weight, fruit yield per hectare, number of dry pods, weight of dry pods, average dry pod weight, number of seeds pod, hundred seed weight, seed weight per pod and seed yield per plant under high heritability category value (>60%). Green fruit width had also low H^2 (<30%)**.**

The current study result indicates that most of the traits (14 out of 25) had high values of heritability, which is important for future okra improvement programs for green fruit, seed yield and related traits. It is important to apply some backcross to concentrate these characters in genotypes because a lot of traits appear to be controlled by genes with additive effects. Heritability is a good index of the degree of transmission of the characters from parents to their offspring (Komal *et al.*, 2022). The consistency in the performance of progeny in succeeding generations depends mainly on the magnitude of the heritable portion of the variation. Heritability indicates the possibility and extent to which improvement can be brought about through selection and it may be of broad and narrow senses (Robinson, 1966).

(Temam, Mohamed and Aklilu, 2020) reported heritability values that ranged from 43.46 to 97.34 % with high heritability for days to 50% emergence, 1st and 50% flowering, days to maturity, stem diameter, number of branches per plant, plant height, and number of fruits per plant, fruit length, fruit width and average fruit weight. Moderate heritability for hundred seed weight. (Melaku, Mohamed and Kumar, 2020) also observed high heritability for all studied traits of 25 okra genotypes except moderate heritability for days to 50% emergence, days to first flowering, and fruit length. (YIMAM, 2018) high values of heritability for the majority of the studied traits except days to maturity that had low heritability and moderate habitability estimated for days to 50% flowering and stem diameter. (Muluken, Wassu and Endale, 2016) reported high heritability for all studied traits except low H^2 for internodes length, moderate days to emergence, number of internodes, number of seeds per pod, and hundred seed weight. (Mihretu, Weyessa and Adugna, 2014) also reported high heritability estimates for fruit diameter, fruit weight, plant height, number of branches per plant, and fruit length.

4.3.2.2. Estimates of genetic advance

In this study, genetic advance percent mean (GAM) ranged between 10.39 to 83.53%. Genetic advance as percent mean (GAM) was categorized as low (0-10%), moderate (10-20%) and high

(≥20%) (Johnson, Robinson and Comstock, 1955). Accordingly, high GAM values were estimated for days to maturity, plant height, all green fruit characters except green fruit width, and all seed yield and related traits. Moderate GAM values were estimated for phenological traits (days to 50% emergence, 1st and 50% of flowering), and growth traits, stem diameter, number of internodes, internodes length, leaf length and width and green fruit width (Table 5).

Table 5. Genetic variability components for 25 traits of 21 okra genotypes evaluated at Dire Dawa in 2020.

Traits	Mean	σ^2g	σ^2p	GCV (%)	PCV (%)	H^2	GA	GAM (%)
Days to emergence	10.95	1.04	2.78	9.33	9.33	37.50	1.29	11.79
Days to 1st flowering	53.3	47.92	130.26	12.98	21.40	36.70	8.64	16.22
Days to 50% flower	64.9	79.89	200.08	13.77	21.80	39.93	11.63	17.93
Days to maturity	79.55	167.64	332.09	16.27	22.90	50.48	18.95	23.82
Plant height (cm)	154.4	409.70	516.08	13.10	14.70	79.39	37.15	24.04
Stem diameter (cm)	3.37	0.14	0.27	11.38	15.48	54.04	0.58	17.23
Number of branches	4.02	1.56	1.94	31.13	34.65	80.75	2.31	57.63
Number of internodes	31.4	12.68	21.42	11.33	14.74	59.17	5.64	17.96
Internode length (cm)	4.94	0.20	0.39	9.16	12.72	51.89	0.67	13.60
Leaf length (cm)	19.36	4.51	8.47	10.96	15.04	53.19	3.19	16.48
Leaf width (cm)	23.8	9.21	22.64	12.75	19.98	40.71	3.99	16.76
Number of green fruit	15.12	18.26	20.26	28.26	29.76	90.17	8.36	55.29
Weight of green fruit	299.4	15279.39	17113.78	41.28	43.68	89.29	240.60	80.35
Green fruit length	14.74	14.97	20.50	26.23	30.70	73.02	6.81	46.18
Green fruit width	2.12	0.03	0.13	9.28	17.07	29.57	0.22	10.39
Average fruit weight	35.58	79.49	104.25	25.05	28.69	76.25	16.03	45.07
Fruit yield (tons ha^{-1})	6.23	6.63	7.42	41.28	43.68	89.28	5.01	80.35
Number of dry pods	15.72	17.04	22.70	26.25	30.30	75.05	7.36	46.85
Weight of dry pods	696.3	51977.57	60077.15	32.73	35.19	86.52	436.84	62.73
ADPW (g)	46.75	391.29	425.82	42.30	44.13	91.89	39.06	83.53
NSPP	79.12	408.27	522.47	25.53	28.88	78.14	36.79	46.50
Hundred seed weight	6.06	0.98	1.30	16.32	18.84	75.05	1.76	29.13
SMC (%)	10.11	2.66	4.66	16.13	21.34	57.15	2.54	25.13
Seed weight per pod	5125.	2105282.11	2563387.33	28.30	31.23	82.13	2708.75	52.84
Seed yield per plant	45.02	360.89	395.40	42.19	44.16	91.27	37.38	83.04

ADPW=Average dry pod weight, NSPP=Number of seeds per pod, SMC=Seed moisture content, PCV=phenotypic coefficient of variation, GCV=Genotypic coefficient of variation, H^2=Heritability in broad sense, GA=Genetic advance, GAM=Genetic advance as percent of mean.

High genetic advance as a percent of mean indicates the traits are controlled by additive genes (Panse and Sukhatme, 1957). Heritability estimates along with genetic advances were more useful in predicting the effect of selecting the best individuals (Johnson, Robinson and Comstock, 1955).

It provides better information than each parameter alone and an expression of additive gene action (Das *et al.*, 2012).

(Temam, Mohamed and Aklilu, 2020) reported GAM values that ranged from 9.16 to 109.14 % in which high genetic advance was observed for all studied traits except days to maturity which had moderate GAM and low for hundred seed weight. (YIMAM, 2018) also reported GAM values that ranged from 5.13 to 91.94% and reported high values of genetic advance values for all studied traits except low GAM for days to 50% flowering and days to maturity, while moderate GAM was observed for days to first flowering. (Melaku, Mohamed and Kumar, 2020) reported GAM values that ranged from 7.7 to 65.15% and high GAM values were observed for plant height, stem diameter, number of branches, number of internodes, internode length, leaf length, leaf width, fruit yield per hectare, number of fruits per plant, green fruit weight, fruit width, number of seed per plant and hundred seed weight while he estimated moderate GAM for days to days to first flowering, 50% flowering, days to maturity and green fruit length and low GAM was found for days to 50% emergence.

(Mihretu, Weyessa and Adugna, 2014; Muluken, Wassu and Endale, 2016; Mohammed, Bekele and Kumar, 2017) also reported high heritability and genetic advance for plant height, green fruit weight, and weight of dry pod per plant. In addition, (Mohammed, Bekele and Kumar, 2017) observed high values of heritability and GAM for dry pod weight, number of seeds per pod, and hundred seed weight. All authors concluded that selection of high performing genotypes would be rewarding in traits that exhibited high heritability coupled with high GAM.

3.4. Principal Component Analysis

Principal component analysis of 25 quantitative traits is presented in table 6. This principal component analysis resulted in four principal components (PCA1 to PCA4) with eigenvalues ranging from 1.95 to 8.75. The first four principal components accounted for a varied percentage of total variance that ranged from 7.83 to 35.02% that summed to a total of 74.56% variation. The first four PCAs were retained in the analysis because each PCA had an eigenvalue >1. The PCAs with an eigenvalue of < 1.00 were ignored due to Gutten's lower bound principle that eigenvalues <1.00 should be ignored (Kumar *et al.*, 2014). (Demelie, Mohamed and Gebre, 2015) worked on okra genotypes and reported PCAs with a cumulative contribution in the range of 64.32% to 83%.

The first four PCAs had the largest contribution with PCA1 (35.02%), PCA2 (20.53%), PCA3 (11.16%), and PCA4 (7.83 %.) contribution. The first four PCAs contributed for a total of 74.56% variance. Similar result was reported by (Demelie, Mohamed and Gebre, 2015) who found the first three principal components (PCA1, PCA2, and PCA3) with values of 32.4%, 16.7%, and 8.2%, respectively, contributed more to the total of 57.3% variation. (Amoatey *et al.*, 2015) reported the first, second and third principal components with values of 32.44%, 19.78%, and 9.68% of the total variance, respectively. (Ahiakpa, 2012) reported that the first principal component (PCA1) was 32.44% variance.

According to (Chahal and Gosal, 2002), characters with largest absolute values closer to unity in the first principal component influence the clustering more than those with lower absolute values closer to zero. Therefore, in the present study, the differentiation of the genotypes into different clusters was because of a cumulative effect of a number of traits rather than the large contribution of a few traits. Days to first flowering, stem diameter, leaf length, number of seeds per pod, seed weight per pod, days to maturity, leaf width, and days to 50 % flowering contributed much to PCA1 in the range between 0.836 to 0.917 and average green fruit weight, weight of green fruit per plant, number of green fruits per plant and fruit yield per hectare with PCA2 contribution ranged from 0.885 to 0.937 (Figure 1; Table 6). Number of dry pods per plant (0.803) and internode length (0.70) much contributed to PCA3 and PCA4, respectively (Figure 2; Table 6). This indicated that these traits had much contribution to the variability of okra genotypes that could be used to evaluate genotypes in the future.

Table 6. The first four principal component axes for 25 quantitative traits of 21 okra genotypes evaluated at Dire Dawa in 2020.

Traits	PCA1	PCA2	PCA3	PAC4
Days to 50% emergence	0.264	0.271	-0.022	0.514
Days to first flowering	**0.836**	-0.221	-0.372	-0.020
Days to 50 % flowering	**0.917**	-0.093	-0.224	-0.091
Days to maturity	**0.873**	-0.029	-0.243	-0.043
Plant height (cm)	-0.040	-0.198	0.445	0.572
Stem diameter (cm)	**0.840**	0.217	0.011	-0.059
Number of branches	0.715	0.095	0.065	-0.258
Leaf length (cm)	**0.842**	0.363	0.024	0.032
Leaf width (cm)	**0.876**	0.288	-0.081	0.016
Number of internodes	-0.125	-0.507	0.488	0.007
Internode length (cm)	0.177	0.362	-0.145	**0.700**
Number of green fruits per plant	0.072	**0.904**	-0.038	0.036
Weight of green fruit per plant (g)	-0.218	**0.891**	0.139	-0.057
Green fruit length (cm)	0.048	-0.611	0.033	0.132
Green fruit width (cm)	-0.471	0.694	0.265	0.132
Average green fruit weight (g)	-0.318	**0.885**	-0.052	-0.041
Fruit yield per hectare (t ha-1)	-0.193	**0.937**	0.041	0.074
Number of dry pods per plant	0.056	0.136	**0.803**	-0.404
Weight of dry pods per plant (g)	0.717	0.285	0.222	-0.444
Average dry pod weight (g)	0.620	0.063	-0.544	-0.098
Number of seeds per pod	**0.849**	0.054	0.199	0.179
Hundred seed weight (g)	0.638	-0.136	0.360	0.344
Seed moisture content (%)	0.447	-0.202	0.518	0.357
Seed weight per pod (mg)	**0.855**	0.096	0.125	0.170
Seed yield per plant (g)	0.515	0.052	0.732	-0.269
Eigenvalue	8.755	5.134	2.792	1.959
Contribution (%)	35.022	20.535	11.167	7.838
Cumulative Variance (%)	35.022	55.557	66.724	74.562

PCA1 to PCA4 represented the first principal component axis to the four principal component axes

Biplot analysis was carried out based on the first two PCAs (Figure 1). The genotypes and quantitative traits were shown on a biplot to visualize their associations and differences. The first and second PCAs biplot explained 55.56% of the total variability among the genotypes displaying days to first flowering, days to 50 % flowering, days to maturity, number of internodes, internode length, stem diameter and green fruit length being considered as the most discriminating traits. The genotypes that were positioned on the left top quadrant were associated and characterized by the tallest green fruit width (GFW), highest weight of green fruit (WGFPP), average green fruit weight (AGF) and green fruit yield (FYD). The genotypes demarcated on the right top quadrant

were associated with the highest number of green fruits per plant (NGFPP), longest leaf length (LL), widest leaf width (LW), widest stem diameter (SD), shortest internode length (IL), early in 50% emergence (DE), lowest number of dry pods per plant (NDPPP), lowest seed yield per plant (SYPP), highest number of branches per plant (NBPP), weight of dry pod (WDPP), average weight of dry pod (ADPW), number of seed per pod (NDSPP), and seed weight per pod (SWPP). The genotypes were positioned on the right bottom quadrant characterized by late days to 1st (FFL) and 50% flowering (HFL) and days to maturity (DM), longest green fruit length (GFL), lowest seed moisture content (SMC) and hundred seed weight (HSW). The genotypes positioned on the left bottom quadrant characterized by the shortest plant height (PH) and the highest number of internodes (NI). The genotypes concentrated around the origin had similar genetic characteristics, while the genotypes that were found far from the origin are considered as unrelated genotypes (Gerrano, van Rensburg and Adebola, 2017). Therefore, selection of these genotypes as potential parents would result in successful hybridization to develop heterotic groups in the okra breeding programme (Figure 1).

PCA3 and PCA4 biplot presented in (Figure 2). These PCAs biplots explained 19% of the total variability among the genotypes, displaying that green fruit width, average green fruit weight, and plant height, seed weight per pod, and seed yield per plant were considered as the most discriminating traits. The genotypes that were positioned on the left top quadrant were closely associated and characterized by late day to 50% emergence (DE) and longest internode length (IL). The genotypes demarcated on the right top quadrant were associated with longest plant height (PH), highest hundred seed weight (HSW) and seed moisture content (SMC), lowest fruit yield (FYD), shortest leaf length (LL), narrowest leaf width (LW), narrowest green fruits width (GFW), lowest number of green fruits per plant (NGFPP), lowest seed weight per pod (SWPP) and lowest number of seed per pod (NDSPP). The genotypes positioned on the left bottom quadrant characterized by early attainable days to 1st (FFL) and 50% flowering (HFL) and days to maturity (DM) and lowest average dry pod weight (ADPW). The genotypes positioned on the right bottom quadrant characterized by the highest number of dry pods per plant (NDPP), weight of dry pod (WDPP), highest seed yield per plant (SYPP, highest number of branches per plant (NBPP), smallest weight of green fruit (WGFPP) and average green fruit weight (AGFW). These PCA3 and PCA4 biplots provided important information regarding the similarities as well as the pattern of differences among the okra genotypes and of the interrelationships between traits. The okra

genotypes scattered in all four quadrants on the axis indicated there was a wide genetic variability for the traits studied. Genotypes that overlapped and closer to each other in the principal component axes had similar genetic makeup. However, genotypes which are far from each other could be considered as genetically distinct (Gerrano, van Rensburg and Adebola, 2017).

3.5. Euclidean Distance and Cluster Analysis

3.5.1. Euclidean Distances among Okra Genotypes

The Euclidean distances of all possible 210 pairs of 21 okra genotypes were estimated from 25 quantitative traits and the result are presented in table 7. The Euclidean distances of the genotypes ranged from 2.33 to 12.56 with mean (6.83), standard deviation (1.8), and coefficient of variation (26.46%) (Table 7). The highest Euclidean distance was computed between the genotypes Humera 01 (northern) and 240591 (south-western Ethiopia) 12.56 followed by Humera 01 (northern) and 242443 (western Ethiopia) 11.82, While, the lowest genetic distance was estimated between Kraft and NamdHari (India) 2.33 followed by between 92203 (western Ethiopia) and Dhenu (India) 3.26. Generally, 87 (41.43%) pairs of genotypes had Euclidean distances of 5.03 to 6.83(overall mean of genotypes minus standard deviation), 56 (26.66%) pairs of genotypes had Euclidean distances 6.84 to 8.63 (between mean and overall mean of genotypes plus standard deviation), while 38 (18.1%) pair of genotypes had Euclidean distances >8.63 and 29(13.81%) pair of genotypes had <5.03 Euclidean distances (Table 7). Generally, the genetic distances measured among the okra genotypes collected from Ethiopia had higher Euclidean distances than the introduced. This showed that there is a higher chance of improving better okra growth, green fruit yield and seed yield and related traits through selection and/or hybridization of okra genotypes from different okra growing regions of Ethiopia.

(YIMAM, 2018) reported the genetic distance for 24 okra genotypes and ranged from 1.96 to 11.36 with the mean, standard deviation, and coefficient of variation of 5.85, 1.97 and 33.75%, respectively. She also observed that genotypes collected from different regions of Ethiopia had higher Euclidean distances than the introduced genotypes. (Mohammed, Bekele and Kumar, 2017) Estimated genetic distances of 25 okra genotypes in which the genetic distance ranged from 3.1 to 12.6 with mean 7.2, Standard deviation 2, and coefficient of variation of 27.85. He also reported the highest Euclidean distances were observed between genotypes collected from Ethiopia and introduced, while the lowest genetic distance was estimated between introduced genotypes.

Genotypes collected from different parts of Ethiopia exhibited wide genetic distances than introduced genotypes (Demelie, Mohamed and Gebre, 2015).

Table 7. Euclidean distances of 21 okra genotypes based on 25 quantitative traits evaluated at Dire Dawa in 2020.

	240586	240609	240591	242443	92203	242433	240209	240207	245157	Humera	*Bamaya*
240586		6.820	9.230	9.077	5.718	6.444	5.247	6.889	8.079	8.884	6.972
240609	6.820		8.355	8.064	5.069	5.167	7.286	4.846	6.657	11.021	8.106
240591	9.230	8.355		5.830	6.413	6.991	6.880	6.663	4.652	12.567	9.483
242443	9.077	8.064	5.830		6.519	6.737	7.276	7.702	5.355	11.823	9.182
92203	5.718	5.069	6.413	6.519		3.673	4.386	5.142	5.349	10.115	7.080
242433	6.444	5.167	6.991	6.737	3.673		4.405	5.692	5.060	9.714	6.417
240209	5.247	7.286	6.880	7.276	4.386	4.405		7.127	6.267	8.803	6.722
240207	6.889	4.846	6.663	7.702	5.142	5.692	7.127		5.475	11.632	8.024
245157	8.079	6.657	4.652	5.355	5.349	5.060	6.267	5.475		11.098	7.658
Humera 01	8.884	11.021	12.567	11.823	10.115	9.714	8.803	11.632	11.098		5.950
23793	6.972	8.106	9.483	9.182	7.080	6.417	6.722	8.024	7.658	5.950	
SOH 714	8.794	7.526	6.043	6.588	6.016	6.148	6.797	6.465	3.789	11.547	8.189
SOH 701	7.268	6.226	7.262	7.987	5.582	5.343	6.603	5.384	4.412	9.138	5.666
Dhenu	4.831	5.698	7.135	6.720	3.262	3.666	4.118	6.049	5.097	9.269	6.176
Kiran	6.832	7.503	10.072	9.733	6.618	7.037	7.147	7.160	7.450	8.252	5.334
Kraft	7.180	7.670	8.669	8.670	6.463	6.584	6.672	7.864	6.023	7.253	4.986
Pocha	8.886	9.534	9.391	9.800	8.256	7.764	7.164	9.386	7.013	8.905	5.975
Mithra	9.090	10.239	9.919	10.177	9.135	8.640	8.431	9.650	7.465	8.553	5.792
A.Anamica	5.505	6.703	7.759	8.040	4.089	5.620	4.926	6.069	6.193	9.447	6.412
NamdHari	7.244	8.075	8.332	8.357	6.839	6.948	6.768	7.920	5.539	7.942	5.210
C.Spineless	4.962	6.249	8.896	8.315	3.799	4.132	4.443	6.696	7.071	8.693	6.243

Table 7 *Continued.*

	SOH 714	SOH 701	Dhenu	Kiran	Kraft	Pocha	Mithra	A. Anamica	NamdHari	C.Spineless
240586	8.794	7.268	4.831	6.832	7.180	8.886	9.090	5.505	7.244	4.962
240609	7.526	6.226	5.698	7.503	7.670	9.534	10.239	6.703	8.075	6.249
240591	6.043	7.262	7.135	10.072	8.669	9.391	9.919	7.759	8.332	8.896
242443	6.588	7.987	6.720	9.733	8.670	9.800	10.177	8.040	8.357	8.315
92203	6.016	5.582	3.262	6.618	6.463	8.256	9.135	4.089	6.839	3.799
242433	6.148	5.343	3.666	7.037	6.584	7.764	8.640	5.620	6.948	4.132
240209	6.797	6.603	4.118	7.147	6.672	7.164	8.431	4.926	6.768	4.443
240207	6.465	5.384	6.049	7.160	7.864	9.386	9.650	6.069	7.920	6.696
245157	3.789	4.412	5.097	7.450	6.023	7.013	7.465	6.193	5.539	7.071
Humera 01	11.547	9.138	9.269	8.252	7.253	8.905	8.553	9.447	7.942	8.693
Bamaya	8.189	5.666	6.176	5.334	4.986	5.975	5.792	6.412	5.210	6.243
SOH 714		5.283	6.374	6.486	6.544	6.041	8.302	6.544	5.801	6.986
SOH 701	5.283		5.318	5.441	4.321	6.183	6.033	5.457	4.025	6.406
Dhenu	6.374	5.318		6.450	5.182	7.210	7.407	3.632	5.416	3.669
Kiran	6.486	5.441	6.450		5.107	5.382	7.064	5.095	5.244	5.187
Kraft	6.544	4.321	5.182	5.107		4.890	4.302	5.238	2.331	6.238
Pocha	6.041	6.183	7.210	5.382	4.890		4.932	6.930	4.330	7.566
Mithra	8.302	6.033	7.407	7.064	4.302	4.932		7.349	3.965	8.997
ArkaAnamica	6.544	5.457	3.632	5.095	5.238	6.930	7.349		5.606	4.054
NamdHari	5.801	4.025	5.416	5.244	2.331	4.330	3.965	5.606		6.831
C.Spineless	6.986	6.406	3.669	5.187	6.238	7.566	8.997	4.054	6.831	

The mean genetic distance of each okra genotype to the other 20 genotypes was calculated to have information about the closest and most distant genotypes (Table 8). Depending on the mean Euclidean distance, Dhenu (India) 5.63 followed by SOH 701 (registered commercial varieties) 5.96 and 92203 (western Ethiopia) 5.97 were found the closest to other genotypes, while genotypes collected from south-western, western and northern Ethiopia were the most distant genotypes to others, 240591 (8.02), 242443 (8.09) and Humera 01 (9.53), respectively. Generally, eight genotypes (38.09%) collected from south-western (240586, 240609, and 240591), western (242443), north-western (240207), northern Ethiopia (Humera 01) and two other genotypes introduced from India (Pocha and Mithra) had mean genetic distance of >6.83 (overall mean genetic distances of genotypes). However, only one genotype collected from northern Ethiopia (Humera 01) had the significantly highest mean genetic distance of >8.63 (overall mean genetic distance of genotype + standard deviation) as presented in table 8.

Table 8. Minimum, maximum and mean of Euclidean distance of 21 okra genotypes estimated from 25 quantitative traits as evaluated at Dire Data in 2020.

Genotype	Minimum	Maximum	Mean	SD	CV (%)
240586	4.830	9.230	**7.190**	1.460	20.340
240609	4.840	11.021	**7.340**	1.650	22.530
240591	4.652	12.567	**8.027**	1.830	22.798
242443	5.355	11.823	**8.098**	1.596	19.707
92203	3.262	10.115	5.976	1.792	29.991
242433	3.666	9.714	6.109	1.573	25.750
240209	4.118	8.803	6.373	1.346	21.126
240207	4.846	11.632	**7.092**	1.689	23.816
245157	3.789	11.098	6.285	1.633	25.980
Humera 01	5.950	12.567	**9.530**	1.677	17.592
23793 (*Bamaya*)	4.986	9.483	6.779	1.288	18.999
SOH 714	3.789	11.547	6.813	1.562	22.931
SOH 701	4.025	9.138	5.967	1.246	20.888
Dhenu	3.262	9.269	5.634	1.537	27.288
Kiran	5.095	10.072	6.730	1.445	21.469
Kraft	2.331	8.670	6.109	1.591	26.036
Pocha	4.330	9.800	**7.277**	1.701	23.379
Mithra	3.965	10.239	**7.772**	1.914	24.633
ArkaAnamica	3.632	9.447	6.033	1.437	23.818
NamdHari	2.331	8.357	6.136	1.671	27.234
Clemson Spineless	3.669	8.997	6.272	1.722	27.449

Overall	2.33	12.56	6.83	1.80	26.46

SD= standard deviation and CV= coefficient of variation in %.

The result suggested the presence of a considerable number of distant okra genotypes with others that could be used in crossing program (YIMAM, 2018). Genetic variations are an important feature to get together the diversified goals of plant breeding, including higher yield, resistance to diseases, quality of the yield and wider adaptations (Ali *et al*., 2014).

3.5.2. Clustering of Genotypes

The Euclidean distance matrix of 210 pairs of genotypes estimated from 25 quantitative traits were used to construct dendrograms based on the Unweighted Pair-group method with Arithmetic Means (UPGMA). Accordingly, the 21 okra genotypes were grouped into four distinct clusters. Cluster I consisted 12 genotypes (57.14%) collected from south western (240586 and 240609), western (92203 and 242433), and north western Ethiopia (240209, 240207 and 245157), two registered commercial varieties (SOH 714 and SOH 701), two genotypes introduced from India (Dhenu and ArkaAnamica) and genotype introduced from USA (Clemson Spineless), while cluster II consisted two genotypes collected from south western (240591) western Ethiopia (242443) 9.52%. Cluster III consisted only one genotype (Humera 01) collected from northern Ethiopia (4.76%) and cluster IV contained six genotypes, one genotype collected from northern Ethiopia (*Bamaya Humera)* and other five introduced from India (Kiran, Kraft, Pocha, Mithra and NamdHari) 28.57% (Figure 3, Table 9).

Table 9. Number of genotypes grouped in four clusters, genotypes, and collection region of 21 okra genotypes evaluated at Dire Dawa in 2020.

Clusters	Number of Genotype	Genotypes	Geographic Origin
I	12	240586 and 240609	South western
		240209, 240207 and 245157	North western and western
		92203 and 242433	Western
		Dhenu, ArkaAnamica	India
		Clemson Spineless	USA
		SOH 714 and SOH 701	Registered commercial verities
II	2	240591, and	South western(Akobo)
		242443	Western (Menge)

III	1	Humera 01	Northern (Tigray)
IV	6	23793 (*Bamaya Humera*)	Northern
		Kiran, Kraft, Pocha, Mithra, NamdHari	India

(Mohammed, Bekele and Kumar, 2017) studied 25 okra genotypes of which 11 and 14 genotypes were obtained from other countries and three geographic regions of Ethiopia, respectively. The genotypes were grouped into seven major clusters. He also indicated genotypes from the same countries tend to be grouped in the same clusters. (YIMAM, 2018) studied 24 okra genotypes of which 10 and 14 genotypes were obtained from other countries and from Ethiopia, respectively. The genotypes were grouped into seven major clusters. (Demelie, Mohamed and Gebre, 2015) studied 25 okra genotypes and identified ten major clusters while (Mihretu, Weyessa and Adugna, 2014) reported five divergent groups of 25 okra collections from two regions (Gambella and Asossa) of Ethiopia. (Binalfew and Alemu, 2016) were able to group 50 okra collections into four major clusters which were obtained from four major production regions of Ethiopia.

3.5.3. Cluster Mean Analysis

The mean values of the four clusters for 25 traits (phenology, growth, green fruits and seed yield and related traits) were presented in table 10. The unique features of Clusters III and IV are characterized by the lowest mean values of crop phenology (early on attain, 50% emergence, 1st and 50% flowering, and days to maturity). Clusters I and IV were distinguished by having higher mean values of plant height, leaf length, number of dry pods, and seed yield per plant. Clusters I and II are also characterized by the highest mean values of all growth traits except plant height and leaf length. Clusters I and III were distinguished by having higher mean values of all green fruit related traits (number of green fruits per plant, weight of green fruit per plant, green fruit width, average green fruit weight, and fruit yield per hectare) except green fruit length. Clusters II and IV were characterized by having higher mean values of green fruit length, while Clusters II and III were characterized by lower mean value of seed moisture content. These results suggested the higher chance of developing varieties through selection and further evaluation of genotypes from these two clusters (Clusters I and III).

According to the mean analysis of clusters, selection of genotypes from Clusters I and III is possible to obtain genotypes with the highest green fruits yield and other desirable traits. It is suggested also to make crosses between the two cluster members and genotypes from Cluster I to combine desirable traits in hybrids and searching better performing varieties in subsequent segregating generations. (Mihretu, Weyessa and Adugna, 2014; Demelie, Mohamed and Gebre, 2015; Binalfew and Alemu, 2016; Mohammed, Bekele and Kumar, 2017) also reported some of the clusters constructed by okra genotypes obtained from Ethiopia had higher mean values for desirable traits including fruit yield, seeds per pod and hundred seed weight.

Table 10. Cluster mean values for 25 quantitative traits of 21 okra genotypes evaluated at Dire Dawa in 2020

	Cluster			
Traits	I	II	III	IV
Days to 50% emergence	11.44	10.67	9.33	10.33
Days to first flowering	54.31	73.70	44.70	45.93
Days to 50% of flowering	68.54	83.70	51.70	53.45
Days to maturity	84.76	101.70	64.30	64.31
Plant height (cm)	155.70	140.15	138.17	159.52
Stem diameter (cm)	3.61	3.70	2.76	2.87
Number of branches per plant	4.66	4.50	2.20	2.87
Number of internodes per plant	20.62	21.25	15.20	16.38
Internode’s length (cm)	25.35	28.18	19.16	20.01
Leaf length (cm)	31.00	29.29	28.06	34.02
Leaf width (cm)	5.05	5.09	4.93	4.67
Number of green fruits per plant	16.57	12.05	17.24	12.86
Weight of green fruit per plant (g	312.92	146.99	458.30	262.31
Green fruit length (cm)	13.78	17.03	11.42	16.44
Green fruit width (cm)	2.17	1.58	2.51	2.13
Average green fruit weight (g)	36.76	22.98	53.26	33.43
Fruit yield per hectare (t ha-1)	6.87	3.06	9.54	5.46
Number of dry pods per plant	16.50	11.16	11.33	16.40
Weight of dry pods per plant (g)	780.15	897.85	524.00	490.35
Average dry pod weight (g)	47.70	91.33	46.33	30.08
Number of seeds per pod	91.33	90.30	31.67	58.87
Hundred seed weight (g)	6.36	6.76	2.33	5.84
Seed moisture content (%)	10.70	9.69	3.25	10.18
Seed weight per pod (mg)	5857.41	6521.95	1933.30	3728.06
Seed yield per plant (g)	53.57	35.510	10.66	36.80

4. CONCLUSION

Generally, the research results indicated to make the following conclusion, i) there are wide variations among okra genotypes for fruit yield, seed yield, and yield related traits. ii), Okra genotypes collected from Ethiopia had higher performance for most of the traits including growth, green fruits yield, and seed yield than introduced genotypes. iii), Okra genotypes collected from Ethiopia were more divergent with high genetic distances than the introduced and two registered commercial varieties. These major results suggested the higher chance of developing okra varieties through selection to be made within the okra genotypes collected from Ethiopia and other countries. However, the evaluation was conducted under non-stress with sufficient irrigation water supply conditions at one location. Therefore, it is recommended to conduct the evaluation of genotypes under controlled and random stresses over location and season.

Data Availability

The raw data and additional information are available from the corresponding author upon request.

Conflict of Interest

The authors declare that there are no conflicts of interest.

Acknowledgments

The authors would like to express gratitude to the administrators of Tony farm research substation of Harmaya University and field data collectors for their cooperation.

5. REFERENCE

Adetuyi, F.O., Osagie, A.U. and Adekunle, A.T. (2011) 'Nutrient, antinutrient, mineral and zinc bioavailability of okra Abelmoschus esculentus (L) Moench Variety', *American Journal of Food and Nutrition*, 1(2), pp. 49–54.

Ahiakpa, J.K. (2012) 'Characterisation of twenty-nine (29) accessions of okra (Abelmoschus spp (L.) Moench) in Ghana'.

Ali, A.S. *et al.* (2014) 'Morpho-Agronomic Characterization of Okra (Abelmuscus esculentus L.)', *World Applied Sciences Journal*, 31(3), pp. 336–340.

Amoatey, H.M. *et al.* (2015) 'Genetic diversity studies in 29 accessions of okra (Abelmoschus spp L.) using 13 quantitative traits'.

Arapitsas, P. (2008) 'Identification and quantification of polyphenolic compounds from okra seeds and skins', *Food Chemistry*, 110(4), pp. 1041–1045.

Binalfew, T. and Alemu, Y. (2016) 'Characterization of okra (Abelmoschus esculentus (L.) Moench) germplasms collected from Western Ethiopia', *International Journal of Research in Agriculture and Forestry*, 3(2).

Bizuayehu, D. *et al.* (2017) 'Duration of low temperature storage, clove topping and smoke water on garlic sprouting and seedling vigor.', *Journal of Agronomy*, 16(3), pp. 124–130.

Burton, C.W. and De Vane, E.H. (1953) 'Estimating heritability in tall Festuca (Restuca

arundinacae) from replicated clonal material', *Agron. J*, 45, pp. 1476–1481.

Chahal, G.S. and Gosal, S.S. (2002) *Principles and procedures of plant breeding: biotechnological and conventional approaches*. Alpha Science Int'l Ltd.

Das, S. *et al.* (2012) 'Genetic parameters and path analysis of yield and its components in okra at different sowing dates in the Gangetic plains of eastern India', *African Journal of Biotechnology*, 11(95), pp. 16132–16141.

Demelie, M., Mohamed, W. and Gebre, E. (2015) 'Genetic diversity of Ethiopian okra collections through multivariate analysis at werer, rift valley of Ethiopia', *The International Journal of Science and Technoledge*, 3(8), p. 186.

Gemede, H.F. *et al.* (2015) 'Nutritional quality and health benefits of okra (Abelmoschus esculentus): A review', *J Food Process Technol*, 6(458), p. 2.

Gerrano, A.S. (2018) 'Agronomic performance, nutritional phenotyping and trait associations of Okra (Abelmoschus esculentus) Genotypes in South Africa', *Rediscovery of Landraces as a Resource for the Future*, 69.

Gerrano, A.S., van Rensburg, W.S.J. and Adebola, P.O. (2017) 'Nutritional composition of immature pods in selected cowpea ['Vigna unguiculata'(L.) Walp.] genotypes in South Africa', *Australian Journal of Crop Science*, 11(2), pp. 134–141.

Gomez, K.A. and Gomez, A.A. (1984) *Statistical procedures for agricultural research*. John wiley & sons.

Hill, W.G. and Mackay, T.F.C. (2004) 'DS Falconer and Introduction to quantitative genetics', *Genetics*, 167(4), pp. 1529–1536.

IPGRI, O.D.L. (1991) 'International Crop Network Series 5', *International Board for Plant Genetic Resources (IBPGR), Rome, Italy* [Preprint].

Jain, S.K. and Allard, R.W. (1960) 'Population studies in predominantly self-pollinated species, I. Evidence for heterozygote advantage in a closed population of barley', *Proceedings of the National Academy of Sciences*, 46(10), pp. 1371–1377.

Johnson, H.W., Robinson, H.F. and Comstock, R.E. (1955) 'Estimates of genetic and environmental variability in soybeans', *Agronomy journal*, 47(7), pp. 314–318.

Komal, J. *et al.* (2022) 'Study of variation among the genotypes of okra [Abelmoschus esculentus (L.) Moench]'.

Kornerup, A. and Wanscher, J. (1981) 'Methuen handbook of colour Fletcher', *Fletcher & Son Ltd, Norwich*, pp. 1–252.

Kumar, R. *et al.* (2014) 'Characterization of Cucumber (cucumis sativus) genotypes through principle component and regression analyses', *Indian Journal of Agricultural Science*, 84(6), pp. 765–769.

Lim, T.K. (2012) *Edible medicinal and non-medicinal plants*. Springer.

Manly, B.F.J. (1986) 'Principal components analysis', *Multivariate statistical methods: A primer* [Preprint].

Melaku, A.B., Mohamed, W. and Kumar, V. (2020) 'Variability, heritability and genetic advance in indigenous and exotic okra [Abelmoschus esculentus (L.) moench] genotypes for yield and yield related traits at Dire Dawa, Eastern Ethiopia', *MOJ Eco Environ Sci*, 5(4), pp. 164–169.

Mihretu, Y., Weyessa, G. and Adugna, D. (2014) 'Variability and association of quantitative characters among okra (Abelmoschus esculentus (L.) Moench) collection in south western Ethiopia.', *Journal of Biological Sciences*, 14(5), pp. 336–342.

Mohammed, W. *et al.* (2022) 'Diversity of Okra [Abelmoschus esculentus (L.) Moench]

Genotypes in Ethiopia', *East African Journal of Sciences*, 16(2), pp. 115–132.

Mohammed, W., Bekele, A. and Kumar, V. (2017) 'Characterization and evaluation of okra [Abelmoschus esculentus (L.) Moench] collections in eastern Ethiopia', *Edited and Compiled by*, p. 211.

Mohammed, W. and Burga, S. (2015) 'Evaluation of potato (Solanum tuberosum L.) genotypes for yeild and tuber quality related traits at Lowland, Dire Dawa, Eastern Ethiopia', *Science, Technology and Arts Research Journal*, 4(3), pp. 1–10.

Muluken, D., Wassu, M. and Endale, G. (2016) 'Variability, heritability and genetic advance in Ethiopian okra [Abelmoschus esculentus (L.) Monech] collections for tender fruit yield and other agro–morphological traits', *Journal of Applied Life Sciences International*, 4(1), pp. 1–12.

Naveed, A., Khan, A.A. and Khan, I.A. (2009) 'Generation mean analysis of water stress tolerance in okra (Abelmoschus esculentus L.)', *Pak. J. Bot*, 41(1), pp. 195–205.

Panse, V.G. and Sukhatme, P. V (1957) 'Genetics of quantitative characters in relation to plant breeding', *Indian J. Genet*, 17(2), pp. 318–328.

Reddy, M.T. *et al.* (2012) 'Genetic divergence analysis of indigenous and exotic collections of okra (Abelmoschus esculentus (L.) Moench)', *Journal of Agricultural Technology*, 8(2), pp. 611–623.

Robinson, H.F. (1966) 'Quantitative genetics in relation to breeding on centennial of Mendelism', in *Indian Journal of Genetics and Plant Breeding*. INDIAN SOC GENET PLANT BREED INDIAN AGRICULTURE RES INST, NEW DELHI DELHI …, p. 171.

Sivasubramanian, S. and Madhavamenon, P. (1973) 'Genotypic and phenotypic variability in rice', *Madras Agric. J*, 60(9–13), pp. 1093–1096.

Sneath, P.H.A. (1979) 'BASIC program for a significance test for clusters in UPGMA dendrograms obtained from squared Euclidean distances', *Computers & Geosciences*, 5(1), pp. 127–137.

Steyn, N.P. *et al.* (2014) 'Nutritional contribution of street foods to the diet of people in developing countries: a systematic review', *Public health nutrition*, 17(6), pp. 1363–1374.

Temam, N., Mohamed, W. and Aklilu, S. (2020) 'Agro morphological characterization and evaluation of okra [Abelmoschus esculentus (L.) Moench] genotypes for yield and other variability components at Melkassa, Central Ethiopia', *MOJ Ecology & Environmental Sciences*, 5(2), pp. 80–87.

Toivonen, P.M.A. and DeEll, J.R. (2002) 'Physiology of fresh-cut fruits and vegetables', *Fresh-cut fruits and vegetables. CRC Press, Boca Raton, FL*, pp. 91–123.

Welsh, J.R. (1981) *Fundamentals of plant genetics and breeding.* John Wiley & Sons.

YADAV, R.K. *et al.* (2017) 'Correlation and path analyses for fruit yield and its component traits in okra [Abelmoschus esculentus L. Moench] genotypes', *International Journal of Agriculture Sciences* [Preprint], (13).

YIMAM, F. (2018) 'GENETIC DIVERSITY AND ASSOCIATION OF SEED YIELD AND'.

Figure

Click here to access/download;Figure;Figures.docx

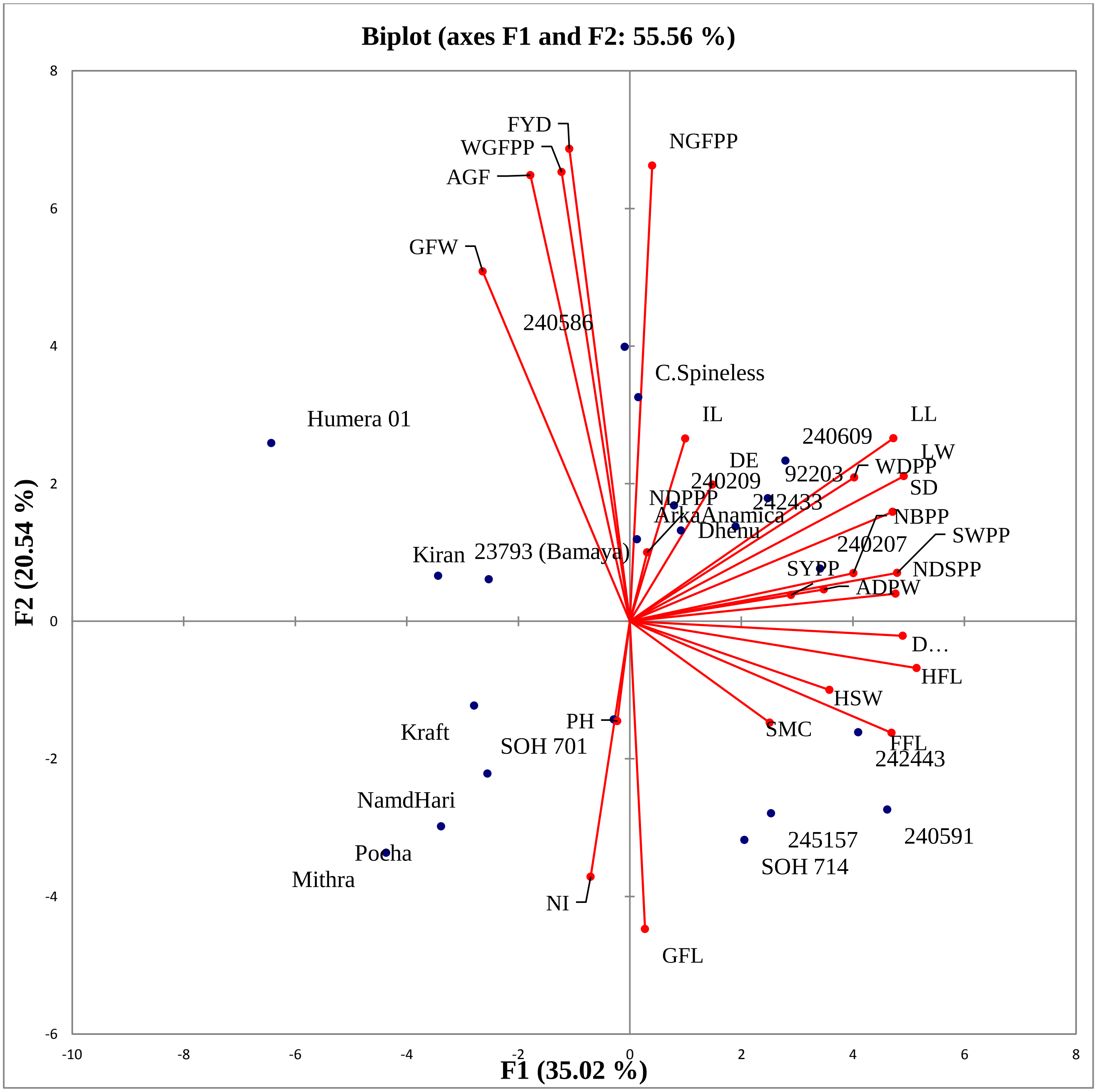

Figure 1. Biplot PCA1 and PCA 2 of 25 quantitative traits of 21 okra genotypes evaluated at Drie Dawa in 2020.

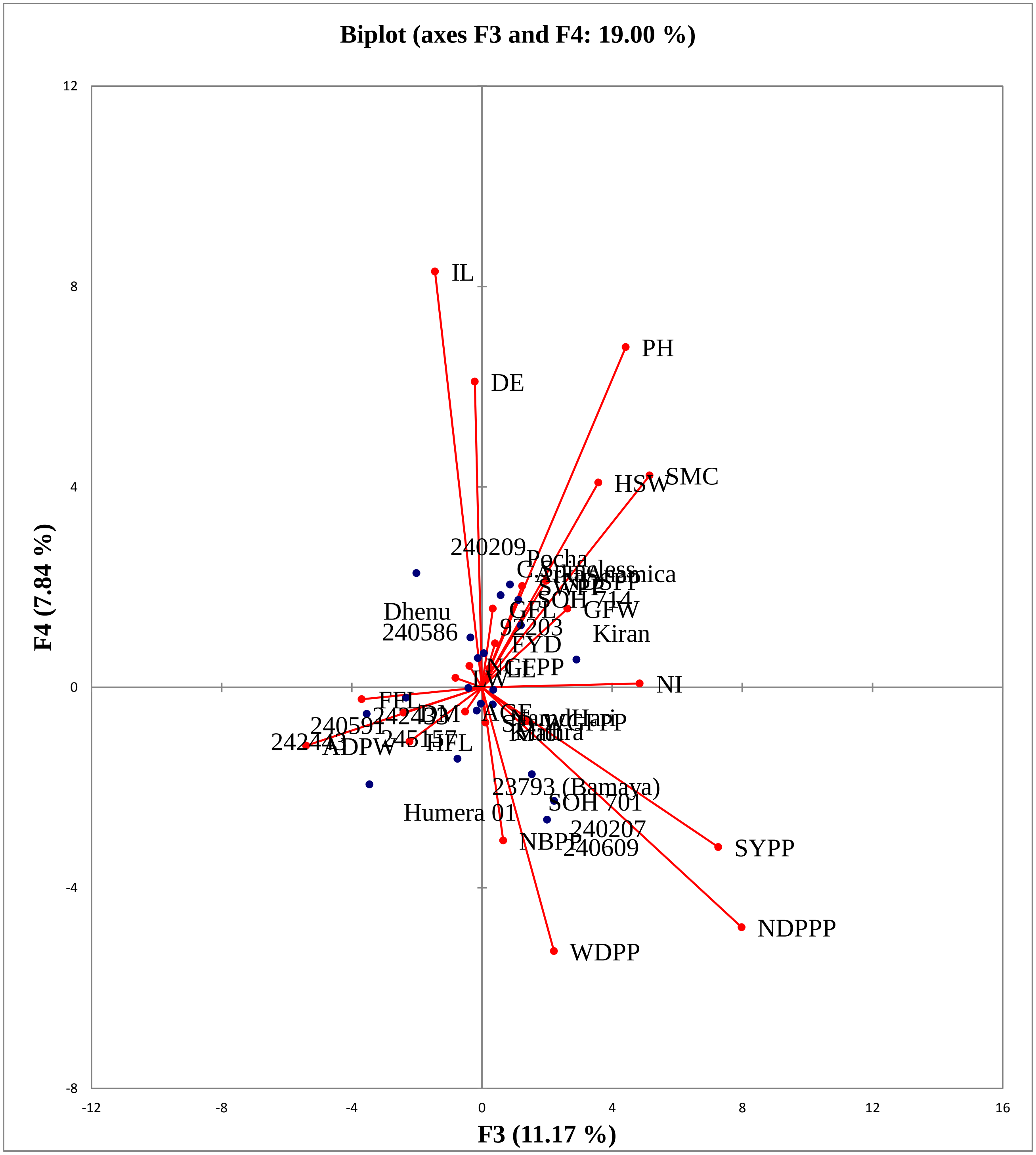

Figure 2. Biplot PCA3 and PCA 4 of 25 quantitative traits of 21 okra genotypes evaluated at Dire Dawa in 2020.

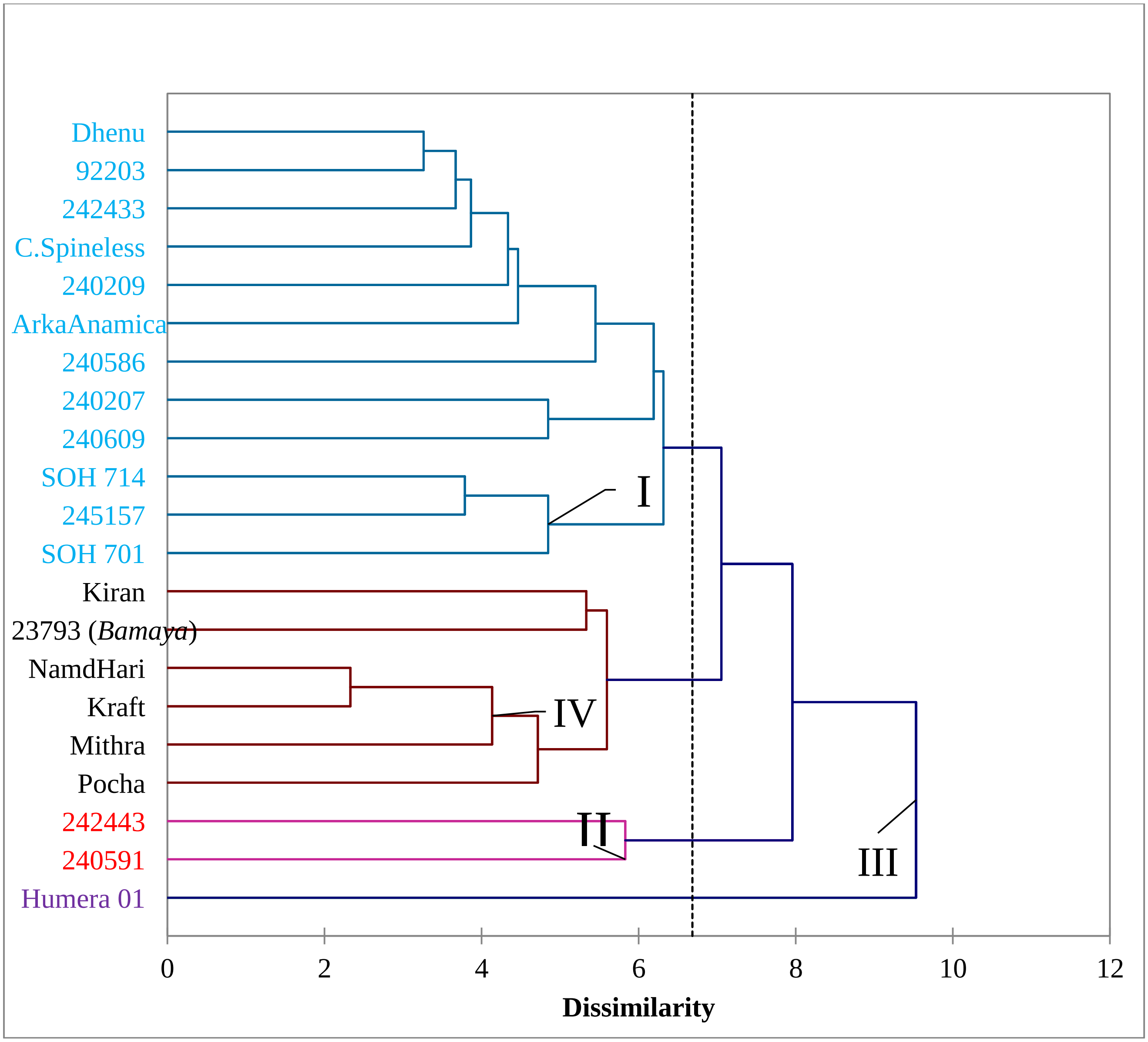

Figure 3. Dendrogram illustrating dissimilarity of 21 okra genotypes by Unweighted Pair Group Method with Arithmetic Means (UPGMA) clustering method with Euclidean distance matrix based on 25 traits evaluated at Dire Dawa in 2020.
