# Supplementary material for: Morpho-agronomic variability of okra [Abelmoschus esculentus (L.) Moench] genotypes in Dire Dawa, eastern Ethiopia
Source: PLoS One. 2023 Jul 14;18(7):e0288534. doi: 10.1371/journal.pone.0288534 (PMC10348553; doi:10.1371/journal.pone.0288534)
Supplement: Attachment — Submitted filename: Response to Reviewers.docx [file pone.0288534.s003.docx]

Author’s response to editor and reviewers

**MANUSCRIPT ID:** PONE-D-23-10456

**MANUSCRIPT TITLE:** Morpho-agronomic variability of okra [*Abelmoschus Esculentus* (L.) Moench] genotypes of diverse origin in Dire Dawa, Eastern Ethiopia

Subject: Revision of the manuscript

We thank the journal and the editors for allowing as the opportunity to publish our work in well reputed journal. We would like to thank the reviewers for the worthy comments. The comments have indeed helped us improve the quality of the paper. We revised the paper extensively and respond in detail to the question and comments for the reviewers. We are open to any further improvements in the paper. Find below the point- by- point description of changes.

| **Editor and Reviewers comment** | **Line number which comment appears** | **Author response** |
| --- | --- | --- |
| The research is a very good one but not concisely and clearly presented. The authors addressed too many things at a time. The manuscript is too lengthy and should be reduced and made clear as much as possible. |  | Thank you for your constructive comment.  We tried to reduce the manuscript to make clear. |
| Although the methods and results of the study is appropriate for a single year/season experiment, but I cannot recommend this article for publication in the Journal (PlosOne) in the present format due to validity of the data and novelty of the work are below the standard level. My major concern is, yield and productivity data (for concluding according to the objectives of this research) should be collected at least for two years/seasons and the experiment must be undertaken in multiple locations. And thereafter, advance statistical analysis (mixed model and multivariate analyses) is necessary to extract data precisely and draw conclusions properly. |  | Thank you for your comment.  This is an interesting question that we are also concerned too. Initially, we proposed that the experiment be conducted at two sites. But, due to the lockdown of the COVID-19 pandemic, the experiment was conducted in only one location. |
| The English language of the article need to be improved considerably. |  | Thank you for your recommendation.  The revised version of main document edited by English language expert person. |
| This cannot be enough reason to justify this study. Give enough problem statement of this study. | Line number 45 | Thank you for your constructive comment.  We addressed the comment in revised version of the main document. |
| This information is not necessary. | Line number 45-49 | Thank you for your comment.  We removed the information from main body as per the reviewer suggestion. |
| Add name of country | Line number 50 | Thank you for your comment.  We added the name of the country in the revised version of document. |
| At what level of significant difference? | Line number 51 | Thank you for your comment.  The traits declared as significant at p value of <0.05 and incorporated the comment in the main body of the document. |
| Provide values of these parameters. | Line number 55-57 | Thank you for your comment.  We incorporated the comments from revised version of documents. |
| Which traits and genotypes were included in these clusters? | Line number 61 and 62 | Thank you for your comment.  We presented the traits in each cluster under “Table 9’’and genotypes under “Table 10.’’ |
| This study has nothing to do with stress tolerance, so why mentioning evaluating to stress tolerance? | Line number 114 and 115 | Thank you for your constructive comment.  This statement was inserted by mistake, now we deleted it from the revised version of document. |
| Growth parameters which include days to germination, plant height, etc. are also morpho-agronomic traits. | Line number 119 | Thank you for your comment.  We corrected the statement based on your suggestion. |
| Correct the tables according given suggestions of the reviewers. | Line number 150 | Thank you for your comment.  We followed journal guidelines and presented the table from the revised version of document. |
| All these descriptions are not necessary. They make the manuscript too voluminous, try to word count of this manuscript. | Line number 204-236 | Thank you for your constructive comment.  We remove the descriptions from the revised version of document as per the reviewer’s suggestion. |
| Using which software and version? | Line number 256 | Thank you for your constructive comment.  We incorporated the comment in revised version of document as per the reviewer’s suggestion. |
| Where is the table that shows result of analysis of variance?  There is no way to ascertain this since the table is not presented. | Line number 323-325 | Thank you for your constructive comment.  We attached the ANOVA table2 and 3 in revised version of document as per the reviewer’s suggestion. |
| This is not the right way of citing reference please. If they are more than two authors, you refer to them as et al. | Line number 327 | Thank you for your constructive comment.  We incorporated the comments by using the journal reference style in revised version of document. |
| All these should be reported under analysis of variance. This manuscript is too lengthy, reporting like this makes the work cumbersome and clumsy. Authors should work on this manuscript by trying to reduce it so as to make it concise, clear and meaningful. | Line number 332 | Thank you for your constructive comment.  Based on the recommendation of the reviewers we reduced the content from the revised version of document. |
| All these traits should be reported once and for all under mean performance of okra. This does not make this work interesting at all. | Line number 352 | Thank you for your constructive comment.  We tried to shorten the contents in revised version of document. |
| Analysis of variance, descriptive statistic, PCA, cluster analysis are enough to describe variability present in a population of crop germplasm so as to avoid ambiguity of information. | Line number 605-727 | Thank you for your constructive comment.  Based on the suggestion of the reviewer we deleted the genetic variability components from revised version of document. |
| So, what is the implication of this value to the variability of the germplasm used?  There is no need of writing all these in the text. This can be seen in the table, simply let us know the value of the first four PCAs and its implication on the variability of the genotypes. | Line number 732 | Thank you for your comment.  We incorporated the comment from main body of document as per the reviewer’s suggestion. |
|  | Line number 737 |  |
| 57.3% is not similar to 74.56% got by the authors. So, authors could have said similar result. Authors should compare their work with authors who obtained value the same or very close to theirs and worked on the same okra. | Line number 739 | Thank you for your comment.  We incorporated the comment in the revised version of main body of document. |
| Which genotypes were found superior?  Which traits were more important? | Line number 922 | Thank you for your comment.  The indigenous okra genotypes were superior in most of the traits and more important than those introduced and incorporated in the revised version of document. |
| Check all references carefully  All references should follow the journal guidelines  Provide DOI for journal references. (PDF file) | Line number 940 | Thank you for your comment.  We use the journal reference guidelines and updated in the revised document. |
| Add latest references in Discussion section. |  | Thank you for your comment.  We included the recent findings that support our work in the revised version of the document. |
| The title should be corrected as "Morpho-Agronomic Variability of Okra [Abelmoschus Esculentus (L.) Moench] Genotypes in Dire Dawa, Eastern Ethiopia" |  | Thank you for your constructive comment.  We corrected the title in the revised version of main document as per the reviewer’s recommendation. |
| What does the alphabets attached to mean values indicate for what information | Line number 446, 504 and 507 | Thank you for your comment.  We indicated the information for alphabets for what was attached with the mean values in the main document. |
|  |  |  |
